# Characterization of bacterial communities of ewe’s vaginal tract and its potential impact on reproductive efficiency

**DOI:** 10.1186/s42523-025-00383-2

**Published:** 2025-05-14

**Authors:** E. L. Reinoso-Peláez, F. Puente-Sánchez, M. Serrano, J. H. Calvo, M. Ramón, M. Saura

**Affiliations:** 1 Instituto Nacional de Investigación y Tecnología Agraria y Alimentaria (INIA-CSIC), Ctra. de La Coruña, km 7.5, 28040 Madrid, Spain; 2 Escuela Técnica Superior de Ingeniería Agronómica, Universidad Politécnica de Madrid, Alimentaria y de Biosistemas, Madrid, Spain; 3 Department of Aquatic Sciences and Assessment, Swedish University of Agricultural Sciences, Uppsala, Sweden; 4 ARAID-Centro de Investigación y Tecnología Agroalimentaria de Aragón (CITA)-IA2, Av. de Montañana, 930, 50059 Zaragoza, Spain; 5 Instituto de Investigaciones Marinas (IIM-CSIC), Rúa Eduardo Cabello 6, 36208 Vigo, Spain

**Keywords:** Artificial insemination, Bacterial communities, Fertility, Ovine, Reproductive success, Vaginal microbiota

## Abstract

The success rate of artificial insemination in sheep remains suboptimal, which has led to an emerging interest in the impact of the reproductive tract microbiome on this process. This research aims to identify the ewes’ vaginal core bacterial community, examine the factors influencing bacterial composition, and to determine the association between vaginal bacteria and pregnancy success. By using a robust dataset comprising 331 multiparous ewes from three Spanish breeds (Latxa, Manchega, Rasa Aragonesa) across four herds, this study performed the sequencing of the hypervariable regions V3-V4 of the 16S ribosomal RNA gene and the identification of Amplicon Sequence Variants (ASV) to analyze the bacterial community. Our analysis revealed a core bacterial primarily consisting of the genera *Streptobacillus, Histophilus, Fusobacterium, Oceanivirga,* and *Parvimonas*. Alpha and beta diversity, as well as Random Forest analysis, identified that herd and breed were the main drivers of bacterial variability. PERMANOVA analysis also showed significant differences in bacterial composition and abundance associated with pregnancy outcomes. Notably, specific ASVs associated with *Fusobacterium*, *Leptotrichia*, *Histophilus*, *Escherichia,* and *Bacteroides* were predominantly found in non-pregnant ewes, while genera such as *Pseudomonas, Acinetobacter,* and *Brevundimonas* were more abundant in pregnant ewes. This study contributes to the knowledge about the critical roles of specific bacteria in determining reproductive success in sheep and provides novel insights about the importance of different factors involved in the composition of ewes’ vaginal bacterial communities.

**Supplementary Information:**

The online version contains supplementary material available at 10.1186/s42523-025-00383-2.

## Introduction

Sheep farming has high economic importance in Spain, which is the second largest sheep producer in Europe and the fifth worldwide [1, 2]. During the last decade, a generalized decline in ovine production has been observed. In particular, Spanish sheep production has decreased from ~20 million animals in 2009 to ~14 million in 2022, representing a 30% reduction [3]. Additionally, European production is not sufficient for its own consumption [4]. Therefore, understanding the factors that negatively affect sheep production efficiency is critical for the sheep production industry.

Artificial insemination (AI) is a key technique in livestock breeding programs, particularly in dairy ruminants [5, 6]. By facilitating the connection of herds, AI enables the comparison of genetic values across all animals and the efficient dissemination of genetic improvement achieved to the whole population [6, 7]. However, the efficiency of AI varies significantly, especially in sheep, with typically lower rates of success that range from 30 to 60% [8, 9]. This fact contributes to a decrease in the economic profitability of farms and slows down the expected genetic gain [10]. Several factors may affect the low fertility rate of AI in sheep. These include the morphology of the ewe’s reproductive tract [11], the requirement of using fresh semen [12, 13], and the difficulty of accurately determining the exact phase of the ewe’s ovulatory cycle at the time of insemination [14].

In the last decade, several studies have evidenced that alterations in the women’s genital microbiota can lead to reproductive dysfunction and even affects sperm motility [15–22].

The key role of the microbiota on the human reproductive tract has laid the basis for microbial investigations in livestock. In bovine, recent investigations have characterized the reproductive microbiota [23, 24] and other research showed that microbial communities can influence reproductive efficiency [25, 26]. Despite the scarcity of studies focusing on ovine reproductive health, there is a growing interest in elucidating the composition and abundance of the vaginal microbiota and its correlation with sheep fertility. In this line, recent studies by Serrano et al. [27], Koester et al. [28], Barba et al. [29], and Reinoso-Peláez et al. [30] identified candidate microorganisms significantly associated with sheep reproductive success. Remarkably, genera such as *Neisseria*, *Oenococcus*, *Mageebacillus*, *Histophilus*, *Actinobacillus*, and *Sneathia* were more abundant in non-pregnant ewes. On the contrary, *Mannheimia*, *Oscillospiraceae*, and *Alistipes* were more abundant in ewes that successfully achieved pregnancy, suggesting that the presence of these taxa may be indicative of a eubiotic state. Furthermore, Serrano et al. [27] and Reinoso-Peláez et al. [30] also showed that Intravaginal progesterone-releasing device (PRID) and synchronization treatments impact microbiota composition, and Greenwood et al. [31] identified significant differences between breeds in the vaginal microbiome. These findings suggest that microbiota composition can be affected by both environmental and host genetic factors.

Under this context, the present study aims to further elucidate the genetic and environmental factors influencing the bacterial dynamics of the vaginal tract potentially impacting reproductive outcomes by AI in sheep by analyzing a dataset of 331 ewes. Our specific objectives were (i) to describe the bacterial core of the sheep attributable to the vaginal tract, (ii) to identify the main factors associated with the composition and abundance of these bacterial communities, and (iii) to determine the composition and abundance of vaginal bacterial communities potentially associated with pregnancy outcome. For this purpose, vaginal samples from different Spanish sheep breeds reared under different production systems and environments were analyzed by amplifying and sequencing the V3-V4 hypervariable regions of the 16S ribosomal rRNA gene. The choice of a substantial sample size not only added robustness to the findings but also reinforced the validity of the study, allowing for a comprehensive analysis across diverse genetic backgrounds and environmental conditions.

## Materials and methods

### Animal samples

**Fig. 1:**
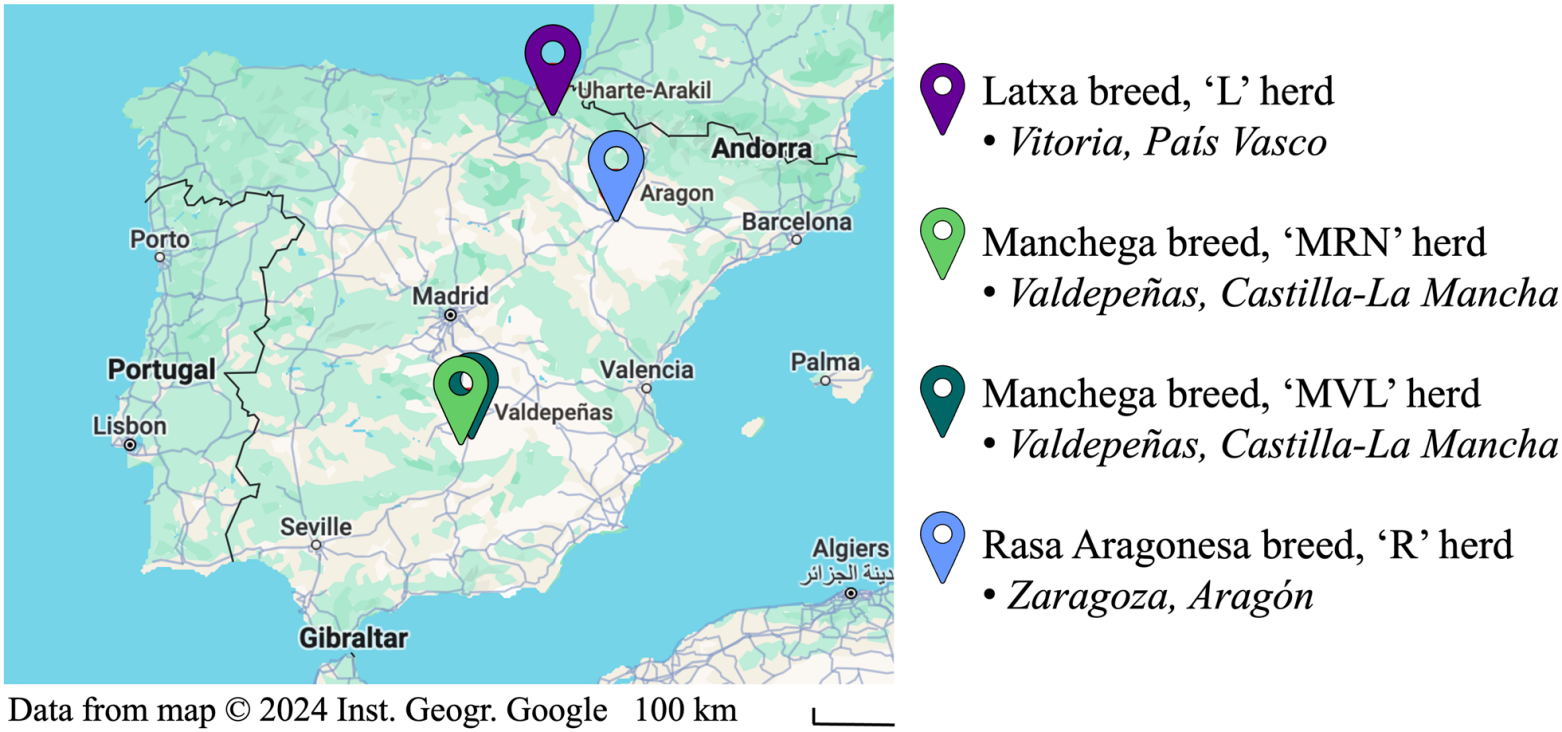
Locations of the farms to which the four herds belonged to

The research involved 331 multiparous ewes, aged between two and five years, and belonging to three breeds reared in four different locations in Spain (Figure 1). It included 71 ewes from Latxa breed (Vitoria, País Vasco; herd L), 119 from Manchega (of which 60 were from herd RN and 59 from herd VL, both from Valdepeñas, Castilla-La Mancha), and 141 from Rasa Aragonesa (Zaragoza, Aragón; we will refer to Rasa, henceforth; herd R).

Ewes were estrous synchronized using a PRID containing 20 mg of Flurogestone acetate (Chronogest MSD Animal Health, Kenilworth, NJ, USA). For Latxa ewes, PRIDs included 0.6 g of powdered Framycetin (neomycin sulfate, Framicas. Laboratorios Ovejero, Spain). No antibiotic treatment was added to the PRIDs used in the other groups. After 14 days, the devices were removed and immediately ewes were injected with a dose of 300–500 mg of Pregnant mare’s serum gonadotropin depending on body weight, to stimulate ovulation. Cervical artificial insemination was conducted 53–55 h after PRIDs removal. Just before insemination, a vaginal exudate was taken from each ewe with a vaginal collection swab (Real Vaginal Microbiome DNA Kit, Durviz S.L., Valencia, Spain). Swabs were immediately refrigerated on dry ice during sampling until arrival to the laboratory, where they were preserved until the extraction at −80 ˚C. Sampling was performed consistently across all groups, using identical swabs and protocols. All samples were collected from the same set of commercial farms by the same trained professional. To avoid cross contamination, we used a speculum to facilitate the sampling, which was disinfected across ewes with povidone-iodine solution in water.

Ewes from each breed were inseminated with fresh semen from 13 Rasa, 12 Latxa and 10 Manchega rams, respectively. Rams used for AI aged between 4 and 7 years and started being semen donors at 10 months of age. The semen underwent minimum quality controls, including mass motility > 3.5 and individual motility > 80/4. Sperm doses were prepared with fresh semen at a concentration of 300 to 400 million of spermatozoids/mL using as diluent INRA96® (IMV Technologies, L’Aigle, France), plus penicillin, gentamicin and amphotericin B and packed in 0.25 mL straws in Rasa and Manchega breeds. In Latxa breed, rams’ sperm doses were prepared with powdered skimmed cow’s milk, plus streptomycin, penicillin, and sodium sulfanilamide.

Pregnancy diagnosis was assessed by transabdominal ultrasound performed between 37 and 55 days post insemination. Fertility was determined based on ultrasound results adjusted for birth outcomes: positive ultrasounds were classified as “positive” for pregnancy, while negative ultrasounds were classified as “negative” unless birth outcomes were positive. This aimed to assess fecundation and the relationship between pregnancy capacity and vaginal bacterial communities.

### DNA extraction and sequencing

Vaginal DNA was extracted with the Real Vaginal Microbiome DNA Kit (Durviz S.L., Valencia, Spain) following the specific protocol for microbial DNA isolation: Swabs were placed in 2 mL microtubes containing 900 µL of CTAB Extraction Buffer and 25 µL of Proteinase K, then incubated at 70 °C for 10 minutes. After bead beating for 10 minutes at maximum speed using a horizontal adapter, samples were centrifuged at 14,000 rpm for 5 minutes. The lysate (900 µL) was transferred to binding buffer (250 µL) and loaded onto silica-membrane spin columns. Following washing steps with Desinhibition and Wash Buffers (500 µL and 700 µL, respectively), DNA was eluted in 100 µL of pre-heated Elution Buffer (70 °C). All centrifugation steps were performed at 14,000 rpm, and the process was completed within approximately 35 minutes. Genomic DNA concentration was measured using a Qubit 4 fluorometer (Thermo Fisher Scientific, DE, USA) and genomic quality ratios (260/280 and 260/230) with Nanodrop 2000 spectrophotometer (Thermo Fisher Scientific, DE, USA).

DNA samples were processed to generate libraries of V3-V4 specific amplicons from bacterial 16S rRNA gene, which were sequenced on Illumina MiSeq using a 2 x 300 bp paired-end run by an external service (Instituto de Parasitología y Biomedicina López-Neyra, Granada, Spain).

### Bioinformatic analysis

Raw sequences were processed using the DADA2 package for R analysis environment [32, 33]. To generate the amplicon sequences of the V3-V4 regions of the 16S rRNA gene the primer pair 341 F (5’-CCTACGGGNGGCWGCAG-3’) and 785R (5’-GACTACHVGGGTATCTAATCC-3’) was used [34]. Primer sequences were trimmed using the Cutadapt software [35]. The selected truncation length for the forward and reverse reads was 240 bp and 200 bp, respectively. The maximum number of assigned errors was set to 2. Bases at the end of a sequence with a quality score ≤ 2 were trimmed. Finally, Amplicon Sequence Variants (ASVs) were identified with the DADA2 algorithm and taxonomically assigned with a Naive Bayesian classifier pre-trained method on the hypervariable regions V3-V4 as implemented in the DADA2 package. The reference database used was the SILVA v132 training set (https://www.arb-silva.de/download/arb-files/; Version: silva_nr_v132_train_set.fa.gz).

### Bacterial composition and diversity analysis

To elucidate the complexity of the vaginal bacterial communities in ewes, the first part of this study focuses on determining the core bacterial community of the sheep vaginal tract, assessing alpha and beta diversities, and the second part aims to conduct a differential abundance analysis to discern taxa variations associated with pregnancy. Additionally, the influence of factors such as breed and herd on bacterial composition was explored, providing a comprehensive overview of the bacterial landscape and its determinants. Three variables were considered for the analyses: (i) breed, with three levels: Latxa, Manchega, and Rasa Aragonesa; (ii) herd*,* that in our data corresponds to the breed, with the exception of Manchega whose samples come from two distinct herds identified by the series codes VL and RN, resulting in four herd levels: Latxa, Manchega VL, Manchega RN, and Rasa; (iii) pregnancy, with two levels: positive or negative. Statistical analyses were performed using R version 4.3.1 [32].

To determine the core bacterial, ASVs were grouped at the genus level, and taxa prevalent in at least 90% of the samples and with a minimum relative abundance (RA) of 2% were selected. The core bacterial was determined for all samples as well as within each level of herd and breed variables.

Alpha-diversity (bacterial diversity within a single sample) was estimated through Shannon index, the number of species weighted by their abundance and evenness of distribution [36]. The Shannon index was preferred as it is better suited for ASV data, providing a comprehensive diversity measure without heavily depending on rare species, unlike Chao1 and ACE index [37]. Alpha-diversity was computed by using Phyloseq R package [38] and rarefaction was performed using the Q10 percentile read count (24,142 reads) to standardize read counts across samples while capturing lower diversity estimates. Statistical significance was assessed using the Wilcoxon rank-sum test.

For the computation of beta-diversity (a measure of dissimilarity between samples based on bacterial communities), the dataset was normalized using the Centered log-ratio (CLR) transformation with the microbiome R package [39], which allows to account for the compositional nature of the data. A Principal Component Analysis (PCA) was conducted with the prcomp function from stats R package [32]. To evaluate differences associated with herd, breed, and pregnancy, we implemented a Permutational Multivariate Analysis of Variance (PERMANOVA) followed by pairwise comparisons between levels of each group. The vegan [40] and RVAideMemoire [41] R packages were used for PERMANOVA and pairwise comparisons, respectively. Differences associated with pregnancy were evaluated for all samples and within each herd.

A cluster analysis was developed to group the samples based on their ASV abundance similarity. For this analysis, the ASV table was filtered by a minimum RA of 1%. To reduce the dimensionality of the data, we applied a Non-Metric Multidimentional Scaling (NMDS) analysis using Euclidean distances (with CLR matrix). Following this, distances were computed using the radial theta algorithm to determine the angle of each data point relative to the centroid of all points in a two-dimensional plane [39, 42], calculated as:

$$\theta =atan2\left(y-\overline{y},x-\overline{x}\right),$$

where *atan2* refers to two-parameter arctangent function, *x* and *y* are the coordinates of a point, and $\overline{x}$ and $\overline{y}$ are the mean coordinates of the NMDS dataset.

Subsequently, the K-means algorithm was applied for clustering the samples under these distances. The optimum number of clusters was selected by the Silhouette Method, which evaluates how similar an object is to its cluster when compared to other clusters. The silhouette scores were computed using the cluster R package [43].

To determine the ability of the identified clusters to predict the analyzed variables (i.e., herd, breed, and pregnancy), Random Forest analysis was conducted. The Random Forest algorithm is a proficient ensemble learning method for classification and estimating variable importance, providing a ranking of each variable based on its significance in prediction. In this analysis, the forest was constructed with a total of 1000 trees with the randomForest R package [44]. To evaluate the performance and precision of the model, a k-fold cross-validation with 5 folds was conducted.

Finally, differential abundance analysis was performed to identify variations in taxa associated with pregnancy across different taxonomic levels, including ASV, species, genus, and phylum using the DESeq2 R package [45] and applying a prevalence filter of 1%. This package employs negative binomial generalized linear models and calculates size factors for data normalization. Additionally, the poscount method was used to deal with the problem of zero-inflated count data. Two approaches were carried out to implement the differential abundance analysis for pregnancy, to which we will refer as: (i) global model, when all samples were considered for the analysis, and (ii) herd-specific model, when a within-breed analysis was performed. To avoid overparameterization and given that the variables breed and herd only differ in one additional level (in the case of herds referred to Manchega breed), the primary variables considered for the model were pregnancy and herd. This decision was validated through Random Forest and AIC tests, confirming that herd was a significant predictor while other factors such as ram effect and birthday had no substantial impact. The global model was represented as:

$$y=X{\beta }+e$$

where $y$ is the vector of abundance of a given taxon; *ß* is a vector that includes the fixed effects of pregnancy and herd in the case of the global model, and only pregnancy in the case of the herd-specific model; *e* is the residual error; and *X* is the incidence matrix relating the observations with the vector of fixed effects.

False Discovery Rate (FDR) multitest correction was applied to adjust p-values at 5% level.

## Results

The fertility rate of all artificially inseminated ewes was 43%, while differences were observed among the different herds: 73% for Latxa, 42% for Rasa, 30% for Manchega VL, and 24% for Manchega RN groups.

### Bacterial composition and diversity analyses

The abundance table included 331 samples, revealing 12,235 ASVs with a total of 12,742,281 reads. The mean read count per sample was 38,496. Taxonomic annotation identified 33 phyla, 67 classes, 161 orders, 351 families, and 906 genera.

**Fig. 2:**
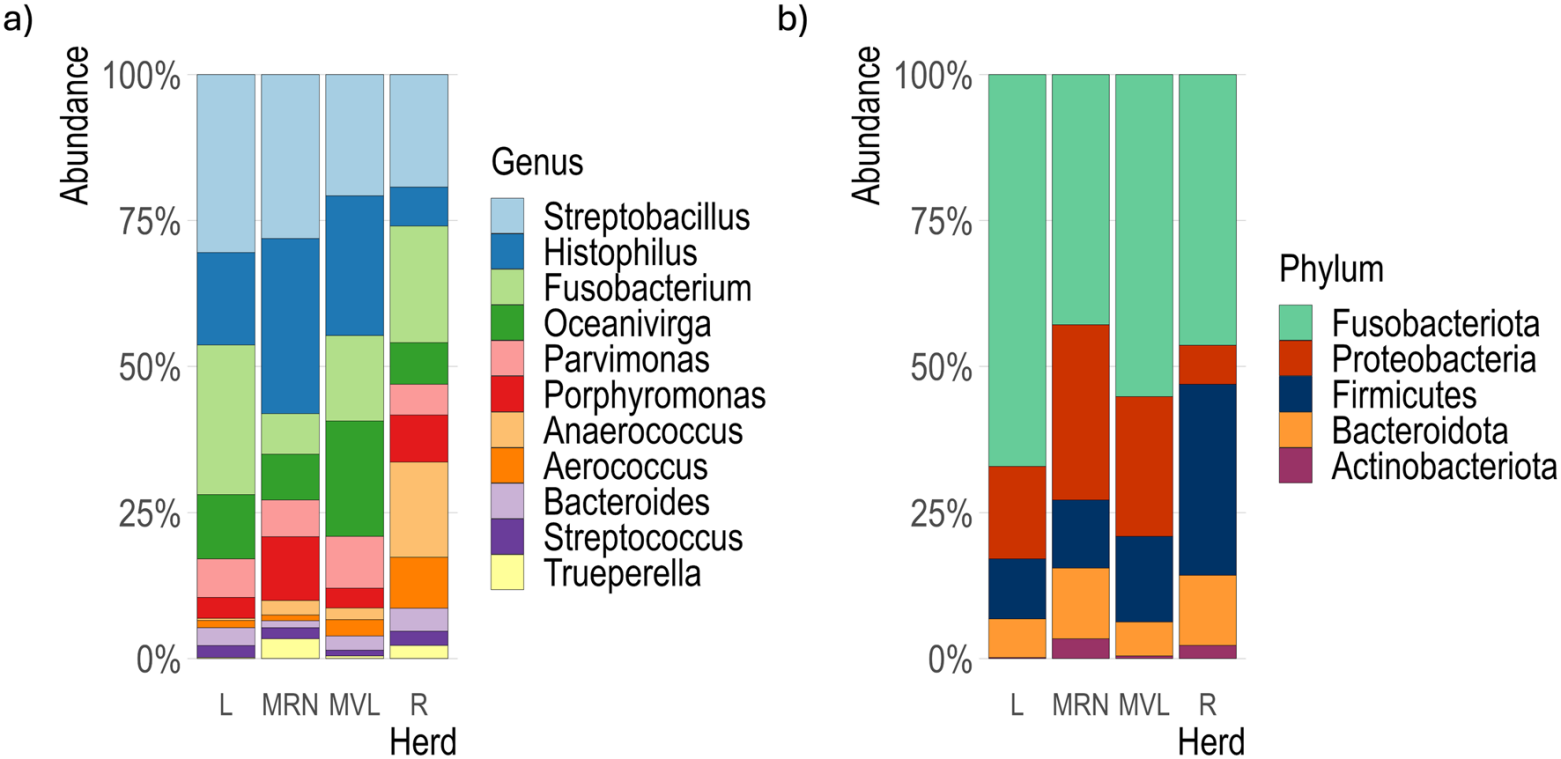
Core bacterial community composition with a prevalence ≥ 90% across all samples, at the genus (**a**) and phylum (**b**) levels, for the four herds analyzed. L: Latxa, MRN: Manchega RN, MVL: Manchega VL, R: Rasa

The core bacterial was integrated by the genera *Streptobacillus* (24%)*, Histophilus* (16%)*, Fusobacterium* (18%)*, Oceanivirga* (10%)*, Anaerococcus* (8%), *Porphyromonas* (7%), *Parvimonas* (6%)*, Aerococcus* (5%), *Bacteroides* (3%)*, Streptococcus* (2%) and *Trueperella* (2%) (Figure 2). The abundance of these genera varied across herd groups; Latxa and Rasa showed a higher abundance of *Fusobacterium*, while Manchega VL and RN herds displayed a greater abundance of *Histophilus* (Figure 2a). This core translated in the following groups and proportions at the phylum level: Proteobacteria (16%), Fusobacteria (52%), Firmicutes (21%), Bacteroidota (10%), and Actinobacteria (2%). The abundance of these phyla varied among herd groups, being Rasa the breed with the lowest abundance of Proteobacteria and the highest abundance of Firmicutes (Figure 2b).

**Fig. 3:**
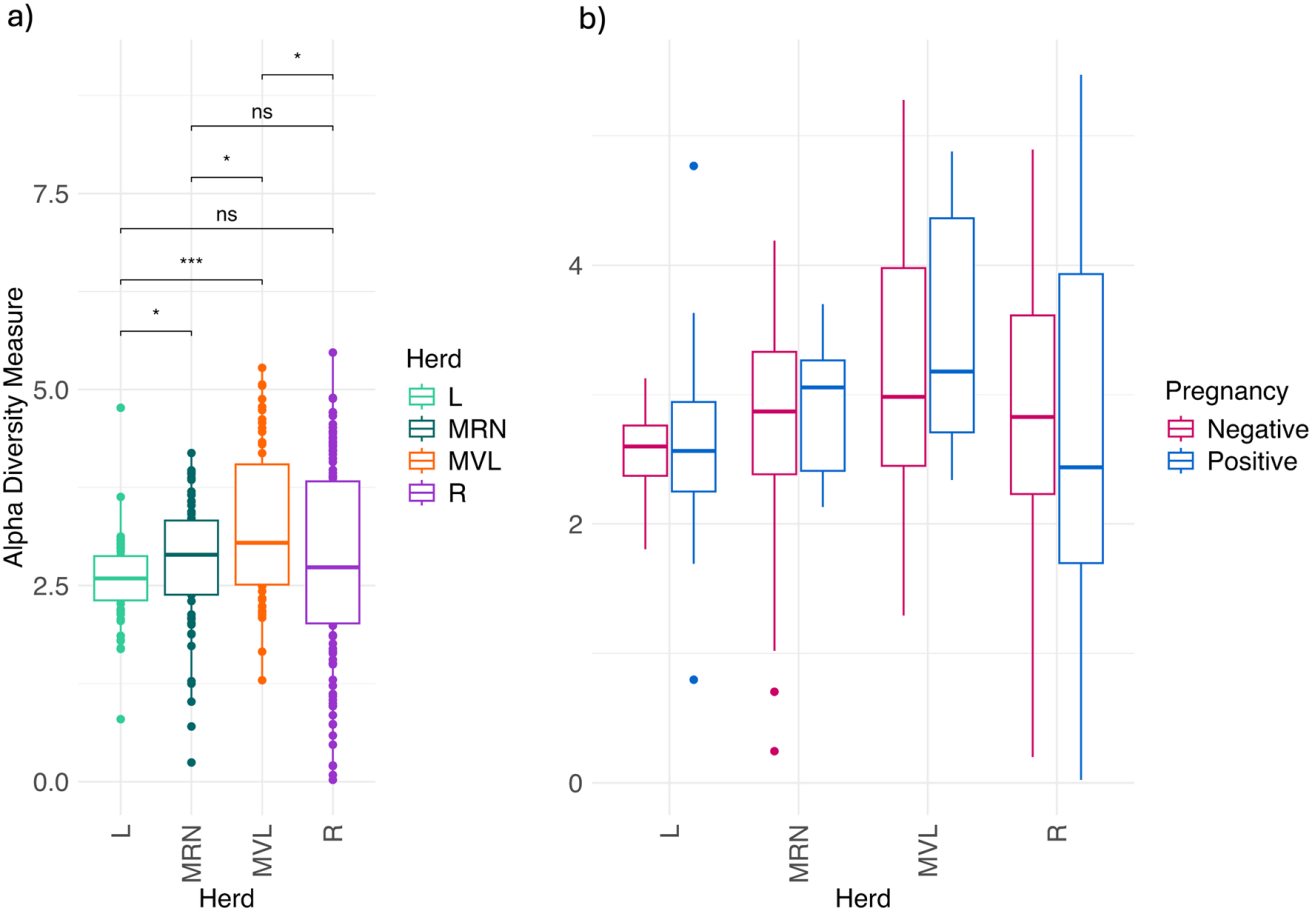
Alpha-diversity (Shannon index) for herd (**a**) and pregnancy (**b**) at the ASV level for the four herds analyzed. Data were rarefied. Statistical significance is indicated by asterisks: * (*p* < 0.05), *** (*p* < 0.001). ns: not significant. Results in Figure b are not significant

Alpha-diversity (Shannon index) varied among herds. Latxa exhibited the lowest diversity, followed by Manchega RN. Manchega VL had the highest diversity, significantly different from the other herds. Rasa displayed more dispersed diversity, with no significant differences compared to Latxa and Manchega RN, but significantly different from Manchega VL (Figure 3a). Although higher Alpha-diversity was observed in pregnant ewes within the Manchega RN and VL herds, no significant differences were found within these groups, among other herds, or across the entire sample set (Figure 3b and Supplementary file Figure S1).

**Fig. 4:**
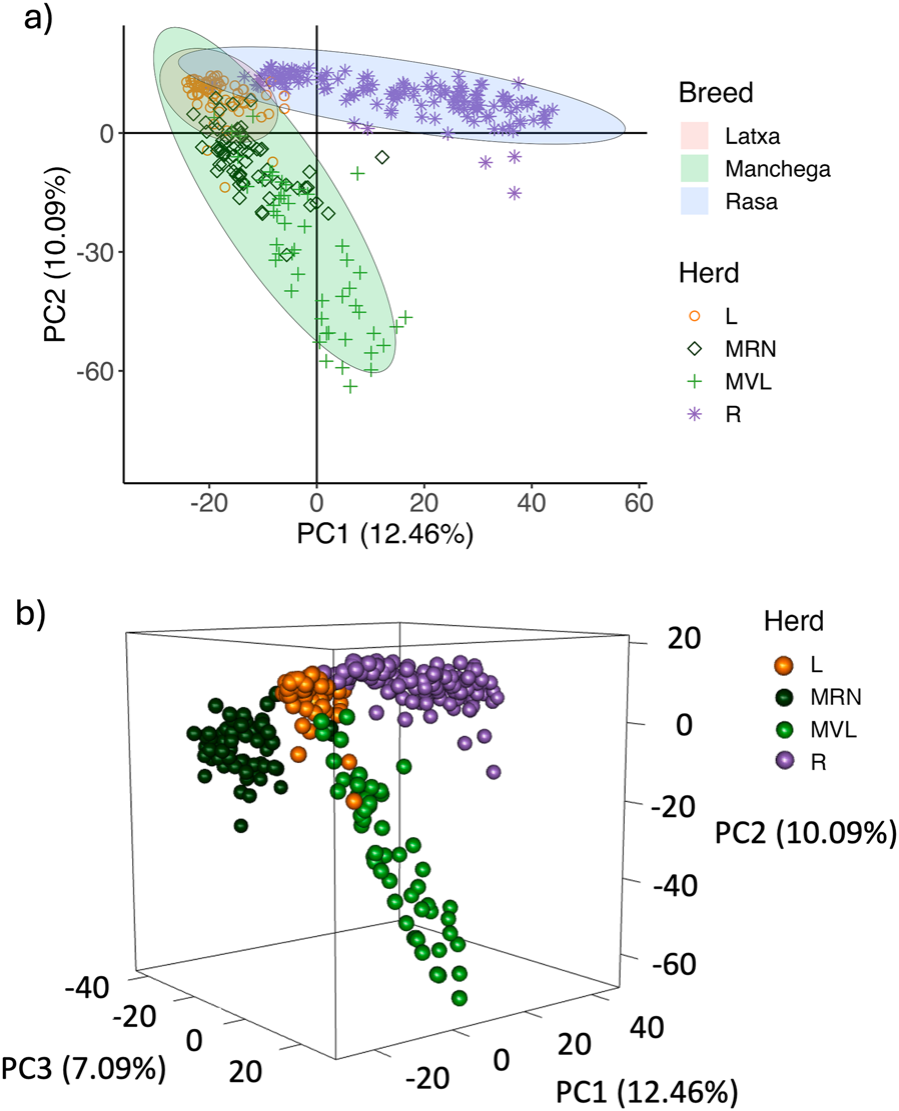
Principal Component Analysis for breed and herd given the bacterial composition at the ASV level, represented in a 2D (**a**) and 3D (**b**) plots. Ellipses in panel (**a**) are calculated using covariance to visualize data variability within each breed group

Principal Component Analysis revealed differences among breeds and herds (Figure 4), with the first three axes explaining a 12.46%, 10.09%, and 7.09% of the total variation, respectively. Latxa and Manchega RN presented a lower dispersion than the other groups. No differences were observed regarding pregnancy status, as shown in Supplementary file Figure S2.

Results from PERMANOVA showed significant differences for herd and breed for the global model, as well as for all pairwise comparisons across all herd groups. Significant differences were also observed for pregnancy under the global model (Table 1).

**Table 1:** PERMANOVA results for breed, herd, and pregnancy variables

	Df	Sum Of Sqs	R^2^	F	Pr (>F)
Herd					
Global	3	207,627	0.2192	30.601	0.001
L-R	1	67,241	0.1214	29.020	0.001
L-MRN	1	52,994	0.2293	40.184	0.001
L-MVL	1	63,942	0.2162	33.661	0.001
R-MRN	1	79,766	0.1357	32.201	0.001
R-MVL	1	78,391	0.1224	26.803	0.001
MRN-MVL	1	63,539	0.2007	29.386	0.001
Breed					
Global	2	144,089	0.1521	29.424	0.001
Latxa-Rasa	1	67,241	0.1214	29.020	0.001
Latxa-Manchega	1	56,198	0.1245	26.752	0.001
Manchega-Rasa	1	87,540	0.1077	31.164	0.001
Pregnancy					
Global	1	5816	0.0061	2.032	0.001
L	1	1019	0.013	0.909	0.633
R	1	2736	0.0067	0.938	0.554
MRN	1	1924	0.0193	1.260	0.131
MVL	1	2705	0.0176	0.916	0.521

*Df*: Degrees of Freedom; Sum Of Sqs: Sum of Squares; R^2^: Coefficient of Determination; F: F-statistic; Pr(>F): *P* value for the F-statistic; MRN: Manchega RN, MVL: Manchega VL.

**Fig. 5:**
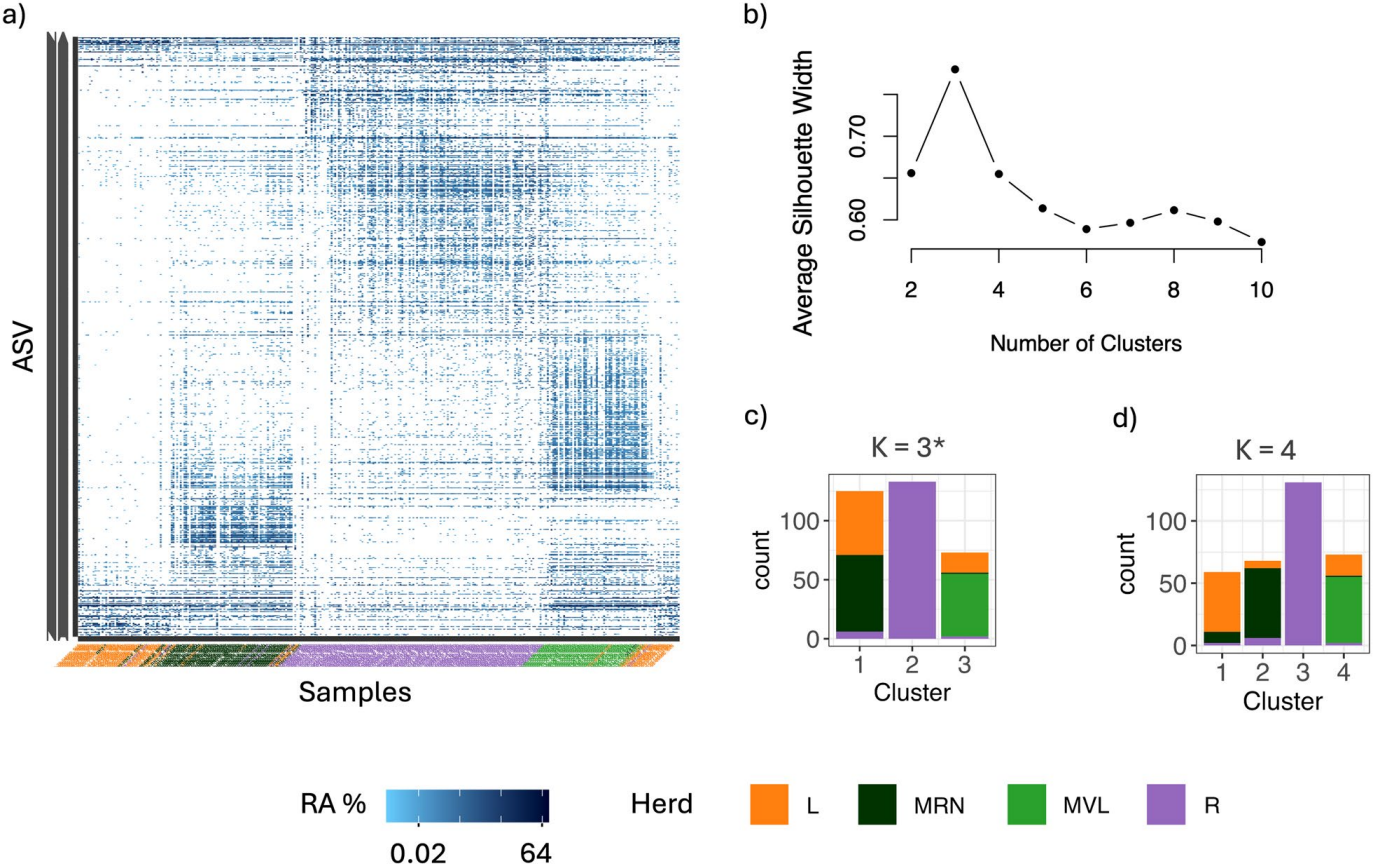
Analysis of sample similarities and clustering in bacterial community studies. (**a**) Heatmap showing the RA of bacterial community composition, arranged according to samples similarity parameters. (**b**) Silhouette width plot for the different numbers of clusters. (**c**) and (**d**) Barplot representing the clusters assuming different number of k. Each bar represents the number of samples within each cluster, color-coded by sample type: L (Latxa), MRN (Manchega RN), MVL (Manchega VL), and R (Rasa). The asterisk (*) highlights the optimal K-value determined by the highest silhouette scores achieved in the analysis

The number of clusters identified by the Silhouette method was k = 3, showing a herd-pattern, with the exception that Latxa and Manchega RN herds clustered together. However, when assuming k = 4, the model followed a clear herd pattern (Figure 5).

The Random Forest analysis revealed that herd was the most important variable for predicting clusters, very closely followed by breed under both RA and CLR normalization approaches. Pregnancy had a negligible effect so far. Cross-validation revealed that the accuracy of prediction for herd was the variable with the highest accuracy (0.89), followed by breed (0.73) (Table 2).

**Table 2:** Assessment of variable importance and predictive accuracy in bacterial community clustering using Random Forest analysis

Variable	^1^Random Forest	
	Mean decrease accuracy	Mean decrease gini
Herd	0.367	100.039
Breed	0.295	68.394
Pregnancy	0.003	3.371

	^2^Cross-Validation	
	Accuracy	SD
Herd	0.8919^a^	0.0177
Breed	0.7285^a^	0.0706
Pregnancy	0.3879^b^	0.0700

Impact of herd, breed, and pregnancy, on bacterial cluster categorization, evaluated by Random Forest importance scores under CLR data transformations. Predictive accuracy was assessed through cross-validation

^1^Random Forest results where the predicted values were the clusters (n = 4) with variables evaluated including herd, breed, and pregnancy. Mean Decrease Accuracy: The average reduction in model accuracy when a variable is omitted. Mean Decrease Gini: The reduction in the Gini coefficient when a variable is omitted, indicating variable importance

^2^Cross Validation: Assesses the predictive accuracy of the Random Forest model via k-fold Method (k = 5). Accuracy: The proportion of correct predictions made by the model, estimated by the mean percentage of those which were correctly assigned. Std: Standard deviation of the accuracy, providing a measure of its variation across the cross-validation folds. Significance between accuracy results is denoted by superscript letters: different letters (a, b, c) indicate statistically significant differences between groups as determined by post-hoc Dunn's testing with FDR adjustment

### Differential abundance analysis

**Fig. 6:**
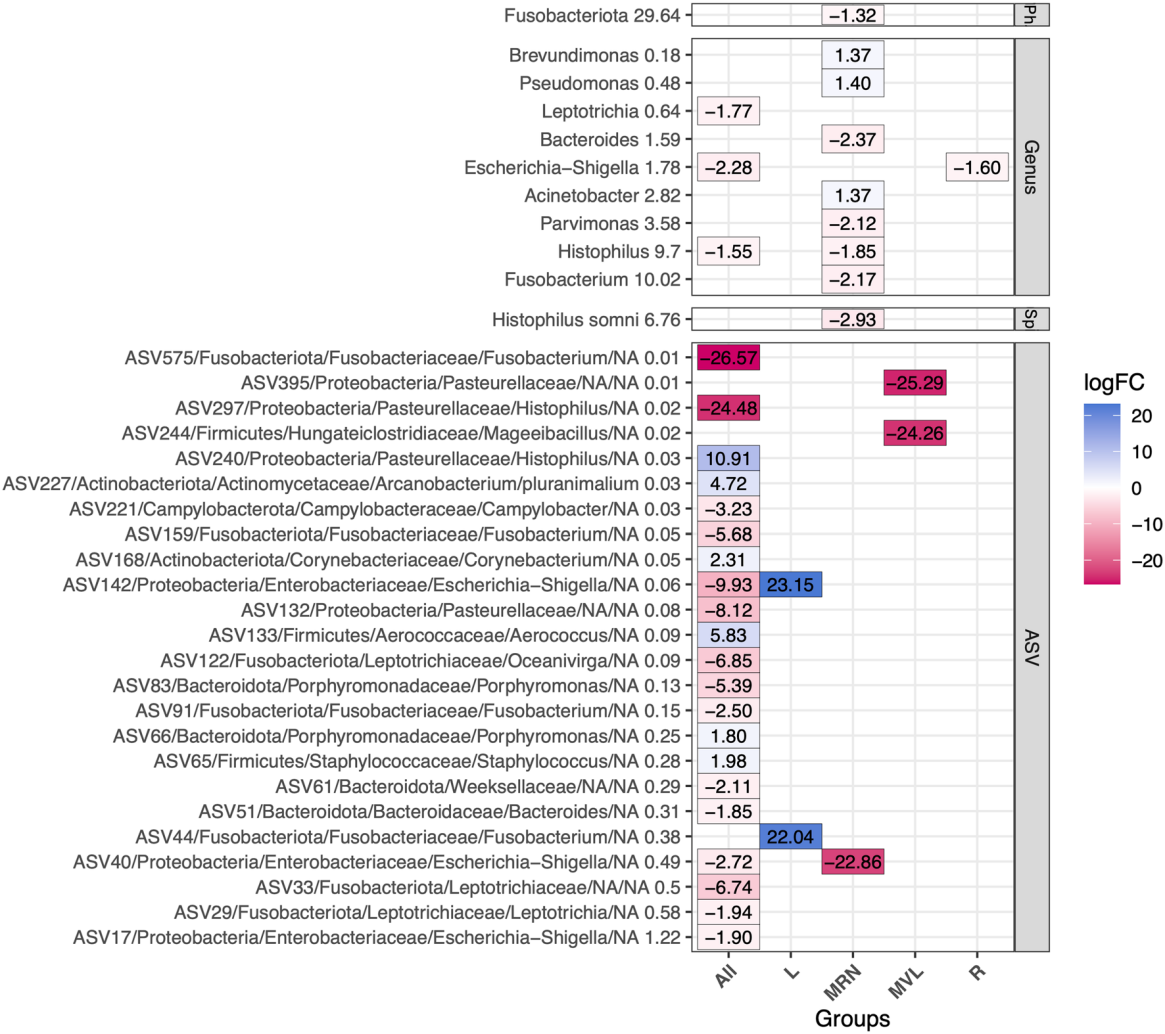
Heatmap showing significant results (FDR at 5%) for differential abundance analysis for pregnancy success at phylum, genus, species and ASV levels. The x-axis shows the results for the global model (ALL) and within each herd (L: Latxa, MRN & MVL: Manchega, R: Rasa). The logFC is represented by color gradations, red for negative and blue for positive on pregnancy success. The color intensity correlates with the logFC value, values close to zero are represented in white. The logFC value is also presented in each square of the heatmap. The y-axis categorizes the taxonomic assignments, delineating a hierarchical classification that includes the ASV code, followed by its corresponding phylum, family, genus, and species, and its RA

Figure 6 summarizes the results from the different analyses performed at the ASV, species, genus and phylum levels. Under the global model, six ASVs presented significantly higher abundance in pregnant ewes, belonging to the genera *Staphylococcus, Porphyromonas, Aerococcus, Corynebacterium, Arcanobacterium,* and *Histophilus,* while fifteen ASVs showed significantly higher abundance in non-pregnant ewes. From the former, thirteen ASVs were attributed to *Escherichia-Shigella, Leptotrichia, Bacteroides, Fusobacterium, Porphyromonas, Oceanivirga, Campylobacter,* and *Histophilus*; and two ASVs attributed to Pasteurellaceae and Weeksellaceae families. When the analysis was performed at the genus level, *Histophilus, Escherichia-Shigella,* and *Leptotrichia* were found to be more abundant in non-pregnant ewes. The distributions of these ASVs are specifically depicted in Supplementary Boxplots in Supplementary file, Figures S6 to S11, providing visual representation of the findings detailed above.

Under the within-herd model there was no consistency across taxa and groups. Hence, Latxa presented two ASVs belonging to *Escherichia-Shigella* and *Fusobacterium* with significantly higher abundance in pregnant ewes, while no taxa were associated with non-pregnant. Contrastingly, Manchega RN presented one ASV belonging to the *Fusobacterium* genus, along with species *Histophilus somni*, and the genera *Fusobacterium, Histophilus, Parvimonas,* and *Bacteroides,* as well as the Fusobacteriota phylum, were significantly more abundant in non-pregnant ewes*.* On the other hand, genera *Acinetobacter, Pseudomonas,* and *Brevundimonas* were found more abundant in pregnant ewes*.* The Manchega VL herd exhibited two ASVs from the genus *Histophilus* and family Pasteurellaceae significantly more abundant in non-pregnant ewes. In line with the global model results, the *Escherichia-Shigella* genus was significantly more abundant in non-pregnant ewes for Rasa.

Additional information of the pregnancy analysis is summarized in Supplementary file, Figures S4–S15.

## Discussion

Our findings revealed that the bacterial core aligns with previous research. showing a high prevalence of the phyla Proteobacteria, Fusobacteriota, Firmicutes, Bacteroidota, and Actinobacteriota, which are key components of the ewe’s vaginal bacterial community. These phyla have also been reported in sheep by Swartz et al. [46], Serrano et al., [27], Greenwood et al. [31], and Reinoso-Peláez et al. [30], as well as in cattle by the work of Swartz et al. [46], Ong et al. [47], and Amat et al. [48]. At genus level, *Streptobacillus*, *Histophilus*, *Fusobacterium*, *Porphyromonas*, and *Bacteroides*, were also identified by Serrano et al. [27] and Reinoso-Peláez et al. [30] even using a different sequencing technique (metagenomics with nanopore). Some of these taxa also agree with the work of Swartz et al. [46], where *Streptobacillus* and *Porphyromonas* were the most abundant genera in Rambouillet ewes from USA. The authors also found *Streptobacillus* in high abundance in cows, suggesting a certain similarity in the bacterial profiles in these two species of ruminants. In contrast, several taxa in our core composition, including *Oceanivirga, Parvimonas, Anaerococcus, Aerococcus, Streptococcus,* and *Trueperella*, were absent in the aforementioned studies, thus suggesting that the vaginal bacterial composition is influenced by both genetic (breed) and environmental factors related to the geographical location, management practices, treatment, and AI, among others. The differing management practices, such as the more extensive systems used for breeds like Latxa and Rasa compared to Manchega, may contribute to these variations. This suggests that our findings, while aligning with existing data, might not be universally applicable across different breeds or management systems due to these inherent differences.

In our study, the Latxa herd exhibited significantly lower Alpha-diversity. This reduced diversity may be associated with the antibiotic treatment applied to the intravaginal sponge for estrus synchronization in this herd. This finding is in line with the study by Reinoso-Peláez et al. [30], who also reported a lower Alpha-diversity in the group of ewes that contained antibiotic treatment in the synchronization sponge. We did not observe a clear association between Alpha-diversity and pregnancy, which differs from previous studies by Chen et al. [49], Serrano et al. [27], Koester et al. [28], and Reinoso-Peláez et al. [30], who suggested that higher Alpha-diversity could be associated with a beneficial effect on pregnancy. Studies in livestock have also highlighted the importance of microbiota diversity in other microbial systems, such as gut and rumen [50, 51], for maintaining animal health and productivity. Conversely, decreases in Alpha-diversity have been linked to various health challenges and production issues [52] probably due to the displacement of certain taxa that form part of the core microbiota, due to an unusual increase of harmful bacteria.

Beta diversity showed a generalized pattern of similarity for individuals of the same herd and breed, according to the results from the PCA. Cluster analysis further demonstrated structured variation in the vaginal bacterial communities across herds. For instance, differences in bacterial clustering were observed depending on the normalization method (RA or CLR) as detailed in Figure 5 and Supplementary file Figure S3. While using CLR reflected a herd-specific clustering pattern, when employing RA, a distinct breed-specific pattern emerged. These variations highlight the impact of normalization matrices on method performance, emphasizing the importance of careful method selection in microbiome studies for reliable biological interpretation. Notwithstanding it is well known that microbiome data are compositional and this needs to be considered. In our study, it is challenging to determine whether herd or breed has a greater influence on the bacterial composition, due to the overlapping between herd and breed according to our experimental design.

Concerning pregnancy, although no significant differences were observed in the global bacterial communities, our PERMANOVA analysis in the global model suggests the presence of subtle patterns and specialized taxa associated with pregnancy success. This is further supported by our differential abundance results, which identified specific taxa linked to reproductive outcomes. Hence, *Fusobacterium* was significantly associated at both the genus and molecular levels (ASV91, ASV159, and ASV575), which showed higher abundance in non-pregnant ewes within the global model and Manchega RN herd (Figure 6, Supplementary file Figures S8, S10, S11, S13). Although these results might suggest a potential influence on reproductive health, *Fusobacterium* was generally less abundant in the RN herd compared to others, challenging simple conclusions about its impact. This genus is known to cause various reproductive disorders [28, 53, 54]. However, given its variable abundance across herds and the lack of consistent associations, the role of *Fusobacterium* in reproductive outcomes in our study appears to be more complex and possibly influenced by herd-specific environmental or management factors, rather than merely its presence or abundance. *Leptotrichia*, also identified at both the genus and molecular levels (ASV29), exhibited a significantly higher abundance in non-pregnant ewes, a pattern consistently observed across all herds (Figure 6, Supplementary file Figures S6, S14. Known as a gram-negative, anaerobic bacteria typically found in the mouth, gastrointestinal tract, and female genital tract [55, 56]. Some species, such as *L. amnionii* or *L. trevisanii* have been linked in humans to spontaneous abortion [57] and fetal demise [58], and to severe acute chorioamnionitis [59], respectively. Although specific species were not identified in this study, these findings allow us to hypothesize about the possible negative effect of this genus on sheep reproductive success. *Histophilus* identified at the genus, species (*Histophilus somni*), and ASV (ASV297) levels, was more abundant in non-pregnant ewes (Figure 6, Supplementary file Figures S11, S12, S13), suggesting its detrimental influence on fertility. This association is supported by findings by Serrano et al. [27] and Koester et al. [28], *Histophilus somni* is implicated in a variety of diseases in cattle and small ruminants, including polyarthritis/tenosynovitis, abortion, fetal septicemia, epididymitis-orchitis, and ocular infections [57, 60, 61]. The genera *Acinetobacter*, *Brevundimonas, and Pseudomonas* displayed a notable higher abundance in pregnant ewes (Figure 6, Supplementary file Figures S13, S14, S15), suggesting their potential relevance in reproductive processes. In humans, Koort et al. [62] found that men with an *Acinetobacter*-associated bacterial community had higher success rates in assisted reproductive technologies, highlighting a possible association. However, further research is needed to confirm its role in ewes. Garcia-Segura et al. [63] reported that *Brevundimonas* inversely correlates with sperm DNA fragmentation and is positively associated with sperm motility and lower oxidative-reduction potential, suggesting its role in improving male fertility. Further exploration and analysis of the species within this genus in our study’s community could be informative. Indeed, the effect of *Acetinobacter* on pregnancy could come in our study from the ram, despite no information about the rams’ bacterial community is available. Conversely, Lennard et al. [64] observed a significant association between *Parvimonas micra* and genital inflammation and persistent bacterial vaginosis in young African females. These findings underscore the importance of further research into the molecular composition of these genera within such populations, providing crucial insights into their effects on reproductive health and emphasizing the necessity for more detailed molecular studies.

At the ASV level, similar ASVs showed divergent impacts on pregnancy, suggesting multifactorial influences. For instance, ASVs with high parity identity (Percentage of identity > 99%, Supplementary file Table S1) to Actinobacillus semini (ASV395 and ASV132), Fusobacterium (ASV44, ASV91, and ASV159), Shigella sonnei (ASV40 and ASV142) —highlighted as an emerging pathogen by Shad and Shad [65] — as well as *Histophilus somini* (ASV240 and ASV297) (Supplementary file Table S1), which previous research by Serrano et al. [27] and Koester et al. [28] has linked to pregnancy disorders, showed contrasting associations in our analysis. This variability highlights the complex interaction between bacterial genetic profiles and pregnancy, evidencing both positive and negative correlations. Notably, the significance of a microbe’s presence is often less critical than its relative abundance, suggesting that minor genetic variations can lead to divergent physiological responses.

An interesting result to highlight is that ASV244 (Figure 6), which corresponds to the genus *Mageeibacillus*, is significantly more abundant in non-pregnant ewes. Although its abundance and prevalence were relatively low (Supplementary file, Figures S4, S5, S11), this microorganism has also been reported in other studies. Serrano et al. [27] reported *Mageeibacillus indolicus* to be less abundant in pregnant ewes and more prevalent in farms with higher artificial insemination failure rates. *Mageeibacillus indolicus*, a recently isolated bacterium from the human vaginal tract, was identified as a key species in distinguishing between full-term spontaneous births and the risk of premature birth [66]. Furthermore, *Mageeibacillus* has been noted as a significant species variable in classifiers used to differentiate between spontaneous full-term births and those at risk of premature birth [67].

Latxa showed two ASVs (ASV44 and ASV142) that were significantly more abundant in pregnant ewes, with no taxa significantly more abundant in non-pregnant ewes. Additionally, the Latxa herd presented the highest pregnancy rate, which could be related to the antibiotic’s effect on potential bacterial groups that may be detrimental to pregnancy. These two ASVs presented very high logFC values. However, an important consideration is that in our results, taxa with the highest logFC values, above |±20| (Figure 6), exhibited low prevalence (<20%) and RA <1% (Supplementary file, Figures S4 and S5). Therefore, it is important to analyze them carefully.

Finally, this study’s large sample size, provides robust insights into bacterial diversity, enhancing understanding and informing future research [68].

## Conclusions

The core bacterial is specific to ewes and likely herd specific. Specific bacterial associations with pregnancy, such as *Fusobacterium, Leptotrichia, Histophilus*, *Escherichia-Shigella,* and *Bacteroides*-related ASVs, were found to be more abundant in non-pregnant ewes, while potentially beneficial genera like *Pseudomonas, Acinetobacter,* and *Brevundimonas* were identified. Importantly, the impact of these taxa on pregnancy appears to be herd-dependent in most cases. Our study suggests that bacterial diversity is mainly influenced by environmental factors, which may include the climatic conditions, the feed provided to the animals, or the management of the ewes. However, an important genetic component is not negligible according to our results. Vaginal bacteria from ewes predicted both herd and breed variables with an accuracy higher than 70%, highlighting the existence of a clear b structure across breeds and herds. The high sample size of this study, exceeding 300 samples, provides robust results that significantly contribute to reinforcement or new insights in the field of bacterial studies. Metagenomic studies in future will be of high value for elucidating specific genes and metabolic networks potentially involved in reproductive failure.

## Supplementary Information

Additional file 1.

## Data Availability

Sequence data that support the findings of this study have been deposited in the Zenodo repository with the DOI: 10.5281/zenodo.12532714
