# Supplementary material for: Characterization of bacterial communities of ewe’s vaginal tract and its potential impact on reproductive efficiency
Source: Anim Microbiome. 2025 May 14;7:48. doi: 10.1186/s42523-025-00383-2 (PMC12079919; doi:10.1186/s42523-025-00383-2)
Supplement: Supplementary file 1 — Additional file 1. [file 42523_2025_383_MOESM1_ESM.pdf]

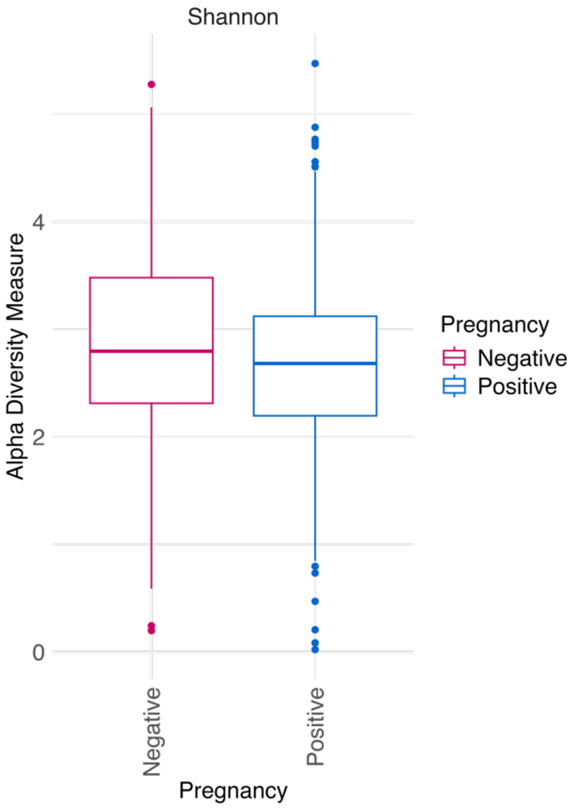

Figure S1. Alpha diversity indices for pregnancy at the ASV level (Shannon index). Data were rarefied.

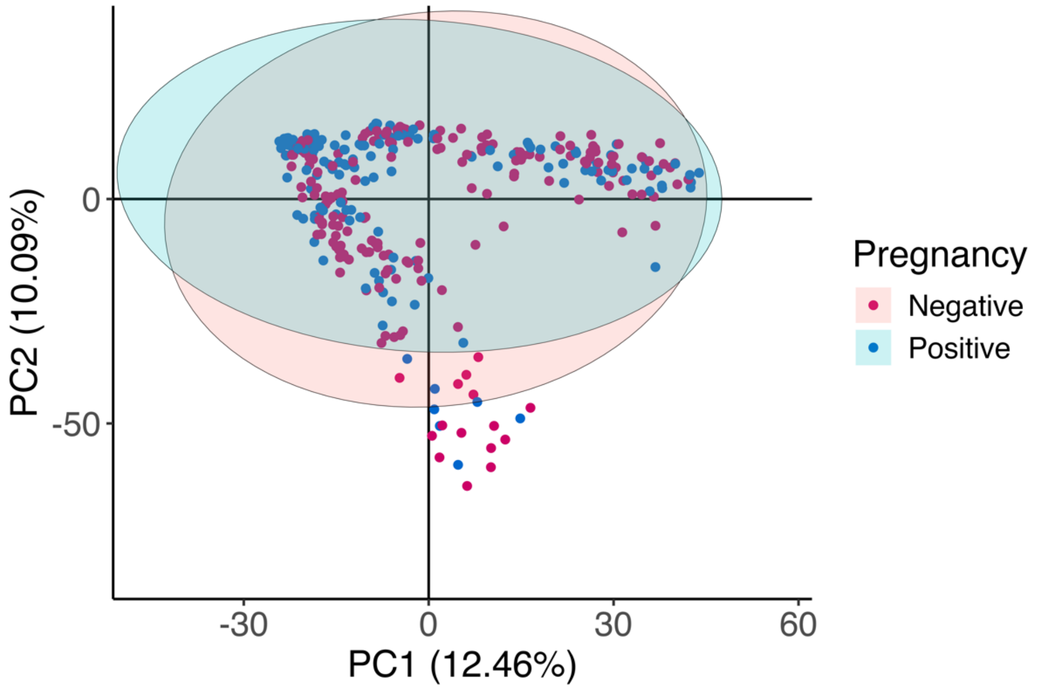

Figure S2. Principal component analysis for pregnancy given the microbiota composition at the ASV level.

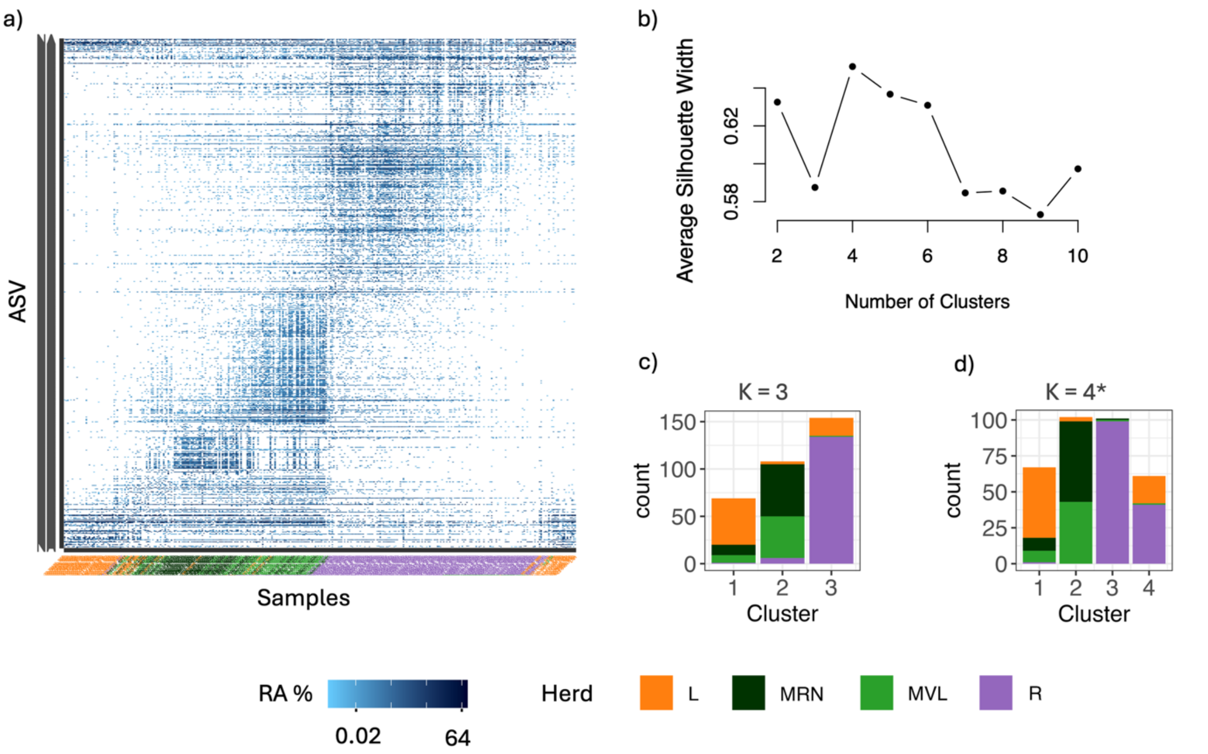

Figure S3. Analysis of sample similarities and clustering in microbial community studies. (a) Heatmap showing the RA of microbial community composition, arranged according to samples similarity parameters. (b) Silhouette width plot for the different numbers of clusters. (c) and (d) Barplot representing the clusters assuming different number of K. Each bar represents the number of samples within each cluster, color-coded by sample type: L (Latxa), MRN (Manchega RN), MVL (Manchega VL), and R (Rasa). The asterisk (*) highlights the optimal K-value determined by the highest silhouette scores achieved in the analysis.

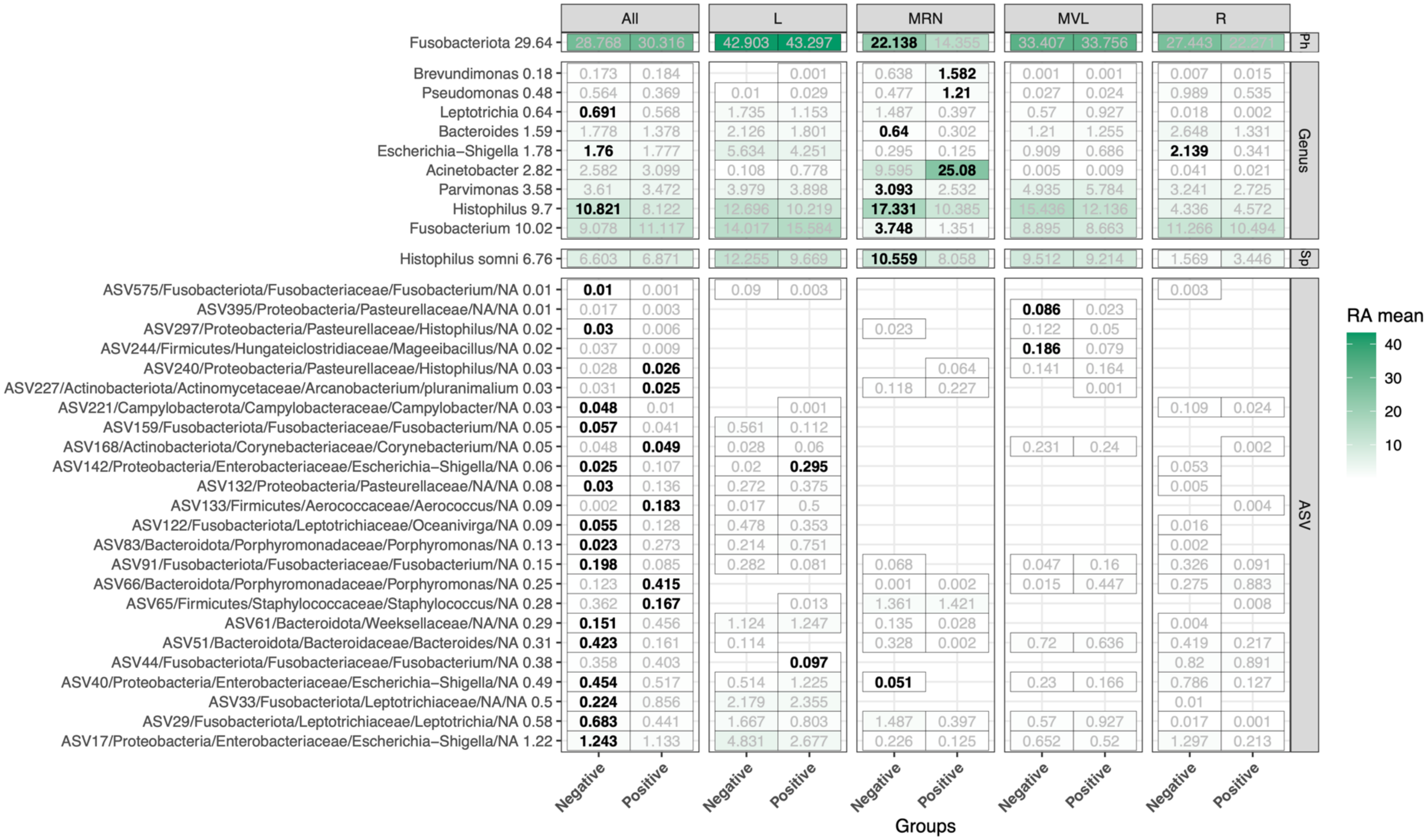

Figure S4. Heatmap representing the RA of taxa against group means (on a 100-point scale) from the differential abundance analysis for pregnancy, for the global model (ALL) and within each herd. Taxa levels include ASV (ASV/code/Phylum/Family/Genus/Species RA), Species, Genus, and Phylum. In bold, significantly differential abundant taxa between pregnant (positive) and non-pregnant (negative) ewes.

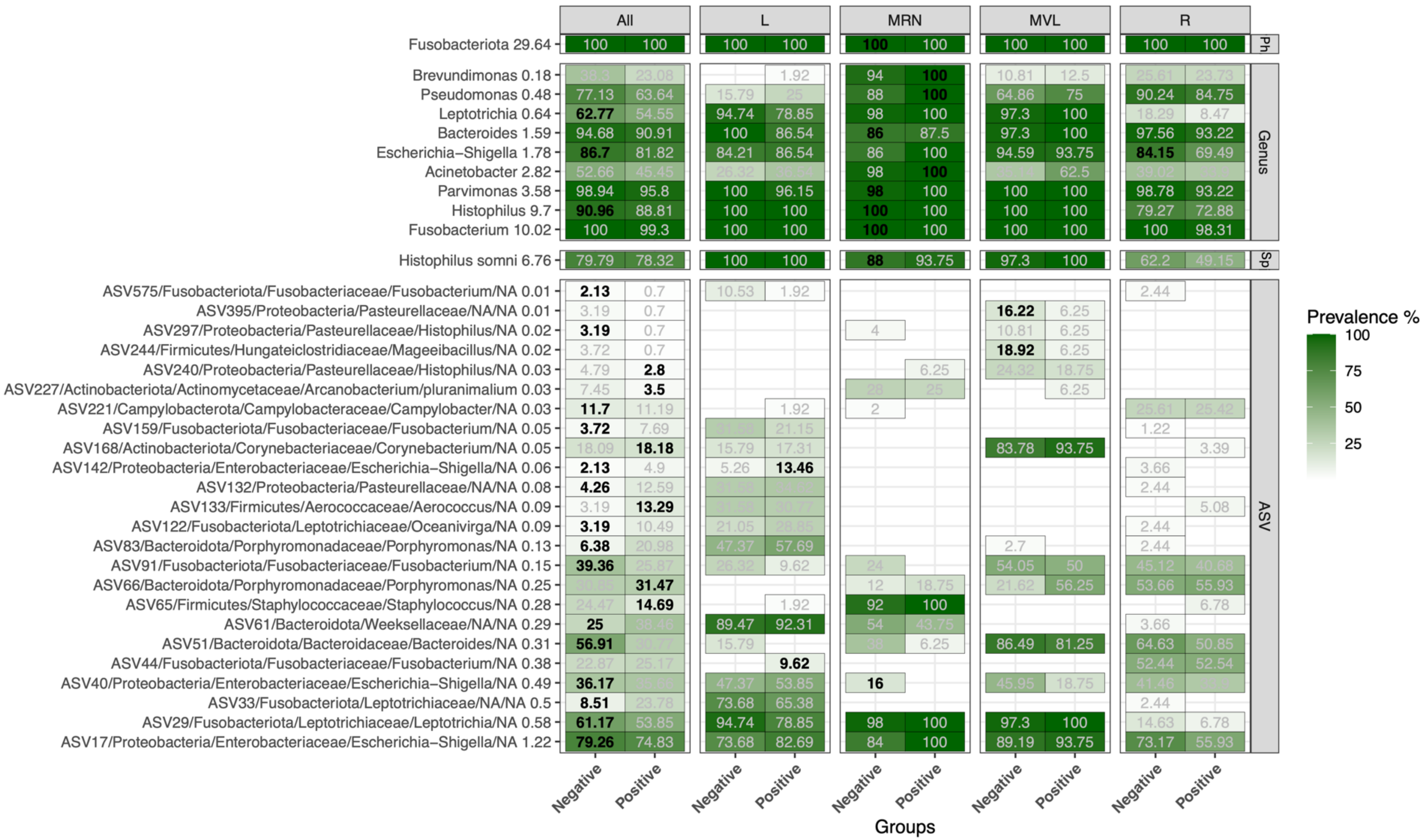

Figure S5. Prevalence of pregnancy by herd groups. Heatmap representation of prevalence of taxa normalized by group on a 100-point scale. Taxa levels include ASV (ASV/code/Phylum/Family/Genus/Species RA), Species, Genus, and Phylum, with significantly abundant pregnancy groups in differential analysis denoted in bold.

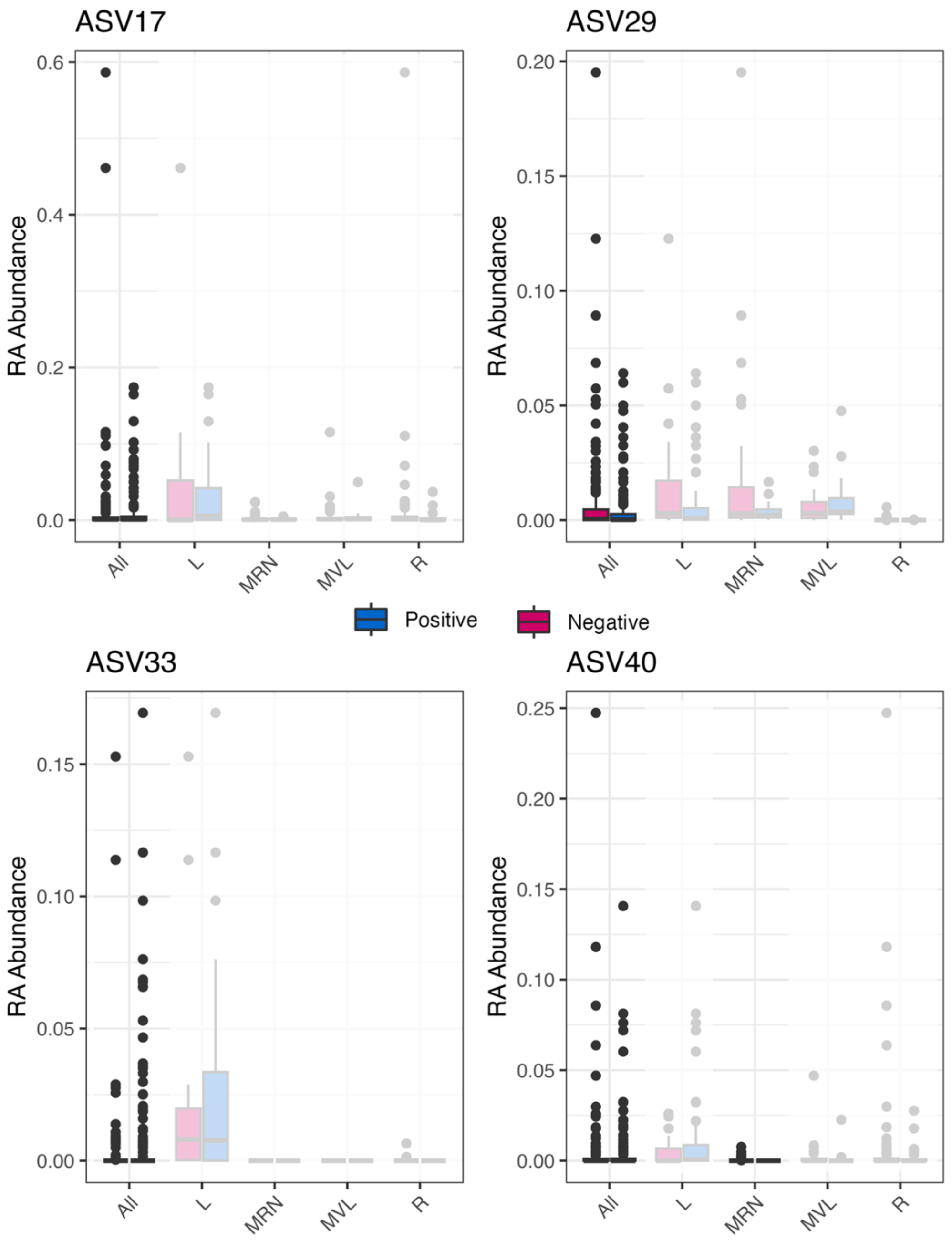

Figure S6. Boxplots of taxa with significant differential abundance between pregnant and non-pregnant ewes for ASV17, ASV29, ASV33, ASV40. Pregnancy-positive samples in blue and pregnancy-negative samples in red. The x-axis categorizes samples by herd level. Saturated colors represent groups with statistically significant differences; desaturated colors are used for groups without significant differences.

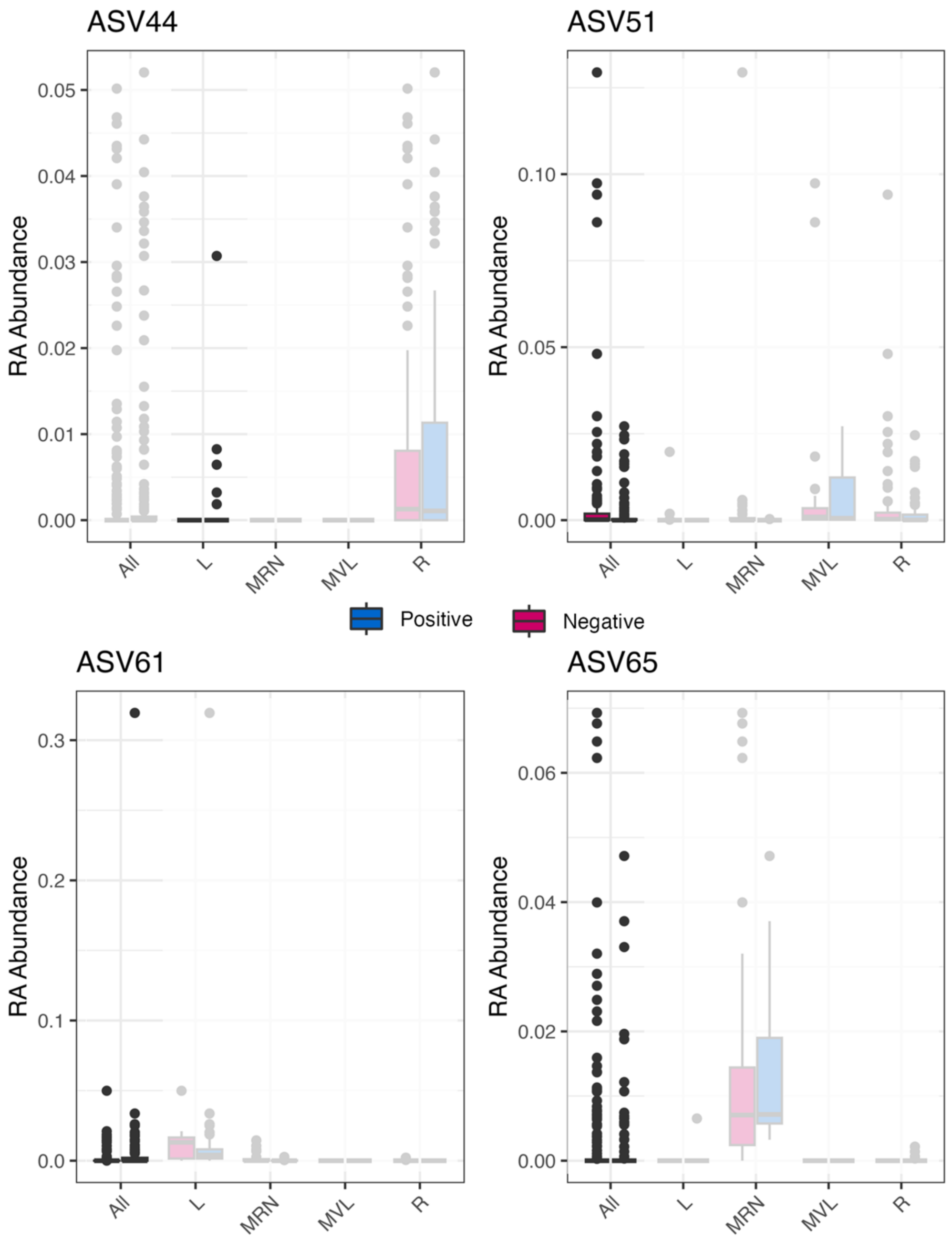

Figure S7. Boxplots of taxa with significant differential abundance between pregnant and non-pregnant ewes for ASV44, ASV51, ASV61, ASV65. Pregnancy-positive samples in blue and pregnancy-negative samples in red. The x-axis categorizes samples by herd level. Saturated colors represent groups with statistically significant differences; desaturated colors are used for groups without significant differences.

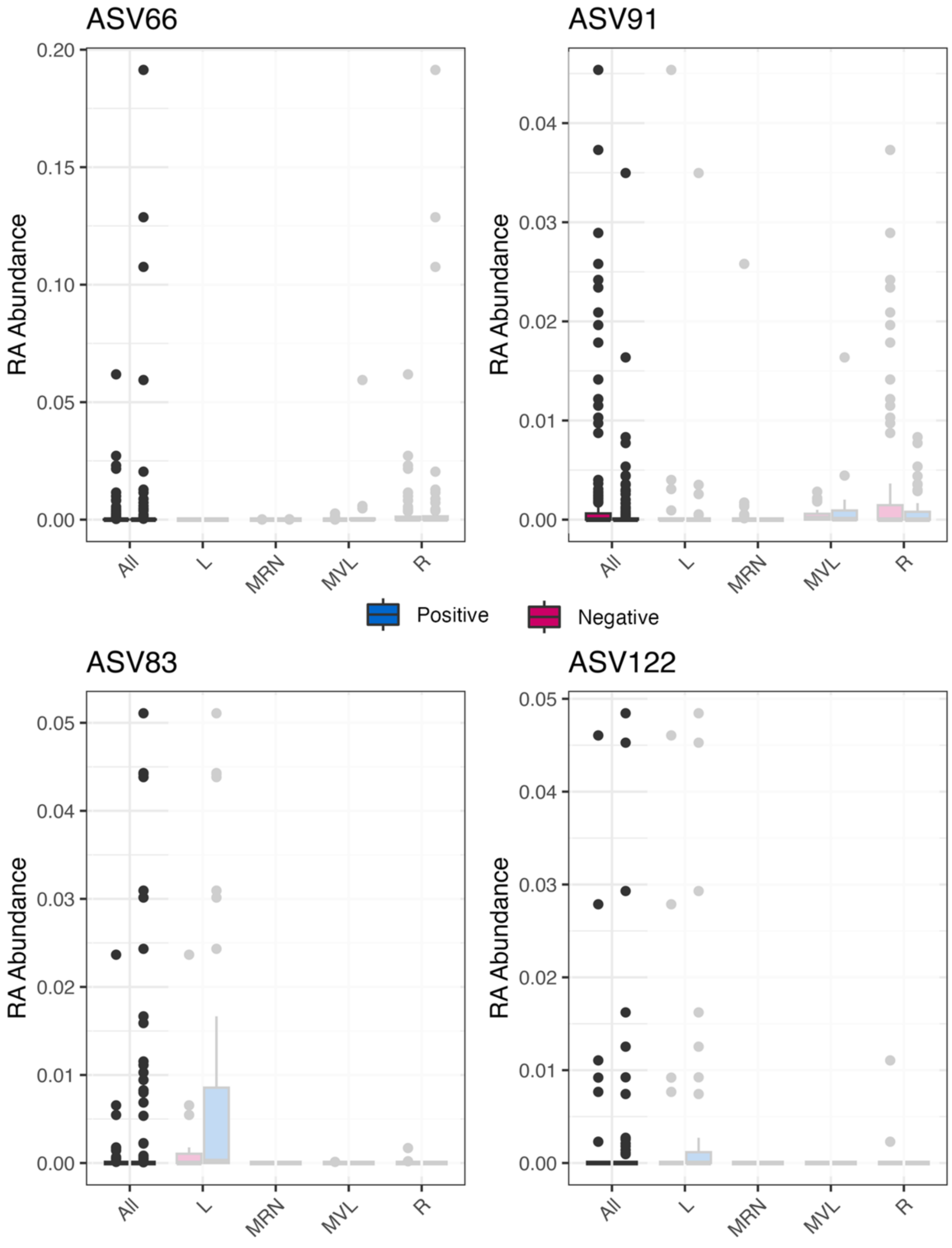

Figure S8. Boxplots of taxa with significant differential abundance between pregnant and non-pregnant ewes for ASV66, ASV91, ASV83, ASV122. Pregnancy-positive samples in blue and pregnancy-negative samples in red. The x-axis categorizes samples by herd level. Saturated colors represent groups with statistically significant differences; desaturated colors are used for groups without significant differences.

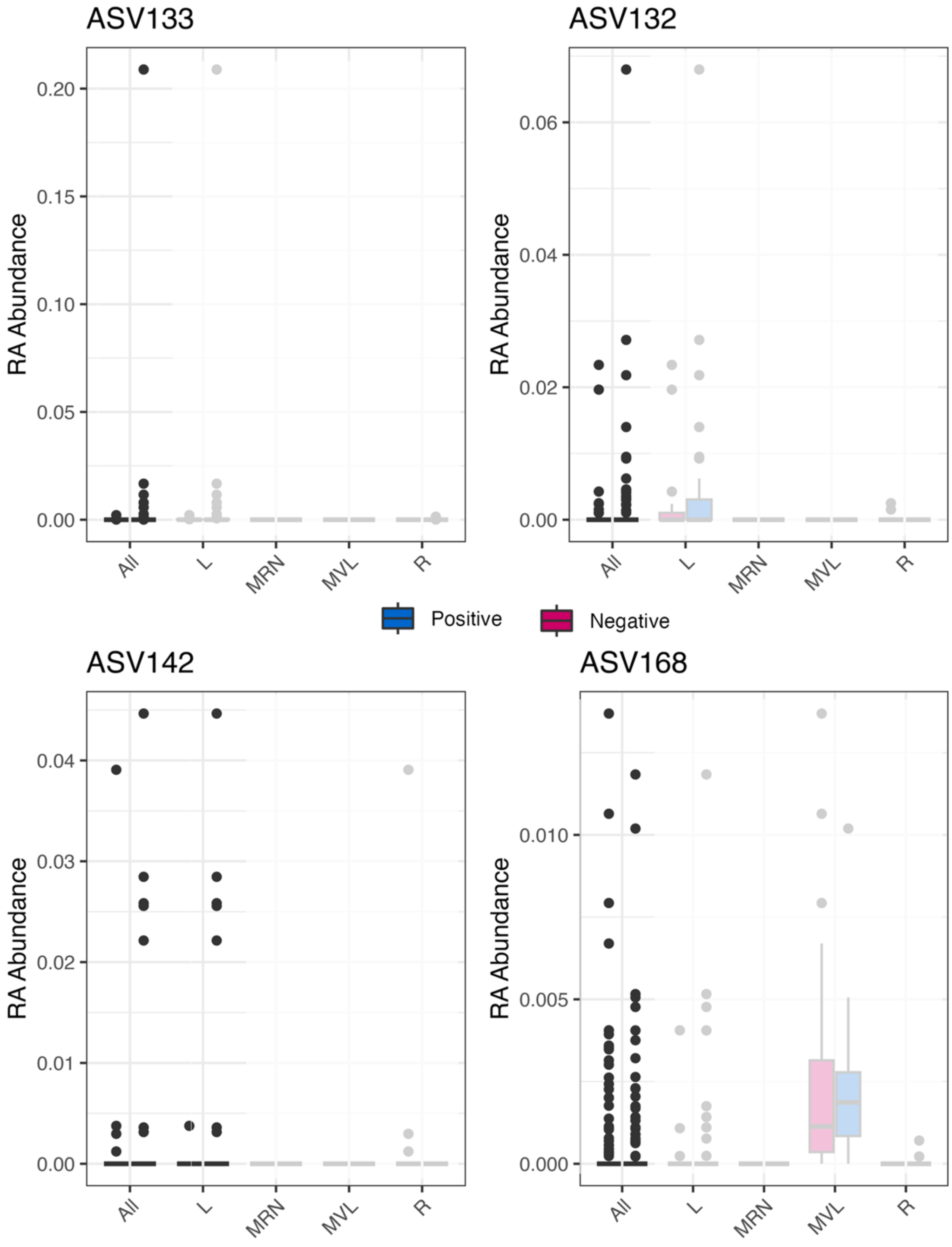

Figure S9. Boxplots of taxa with significant differential abundance between pregnant and non-pregnant ewes for ASV133, ASV132, ASV142, ASV168. Pregnancy-positive samples in blue and pregnancy-negative samples in red. The x-axis categorizes samples by herd level. Saturated colors represent groups with statistically significant differences; desaturated colors are used for groups without significant differences.

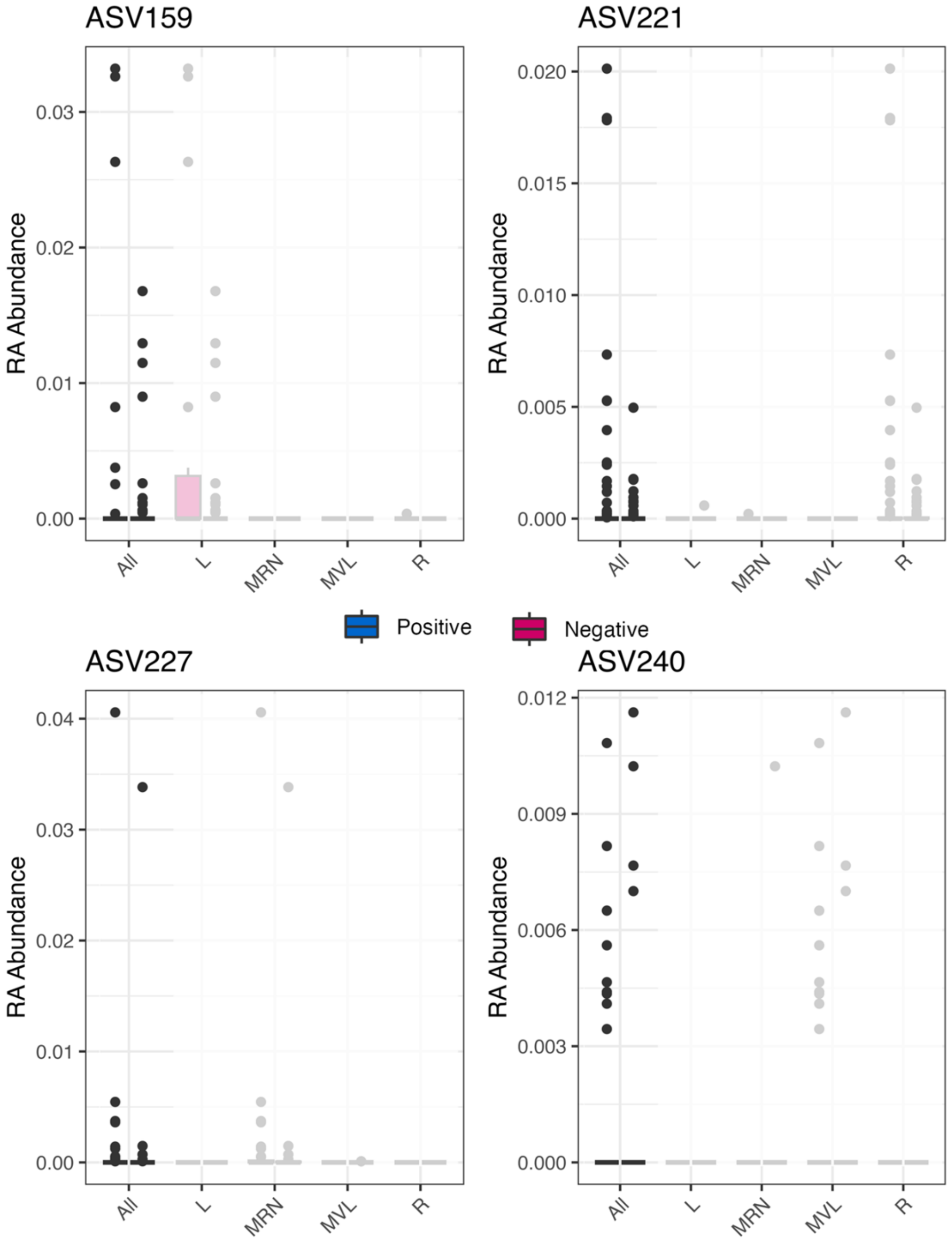

Figure S10. Boxplots of taxa with significant differential abundance between pregnant and non-pregnant ewes for ASV159, ASV221, ASV227, ASV240. Pregnancy-positive samples in blue and pregnancy-negative samples in red. The x-axis categorizes samples by herd level. Saturated colors represent groups with statistically significant differences; desaturated colors are used for groups without significant differences.

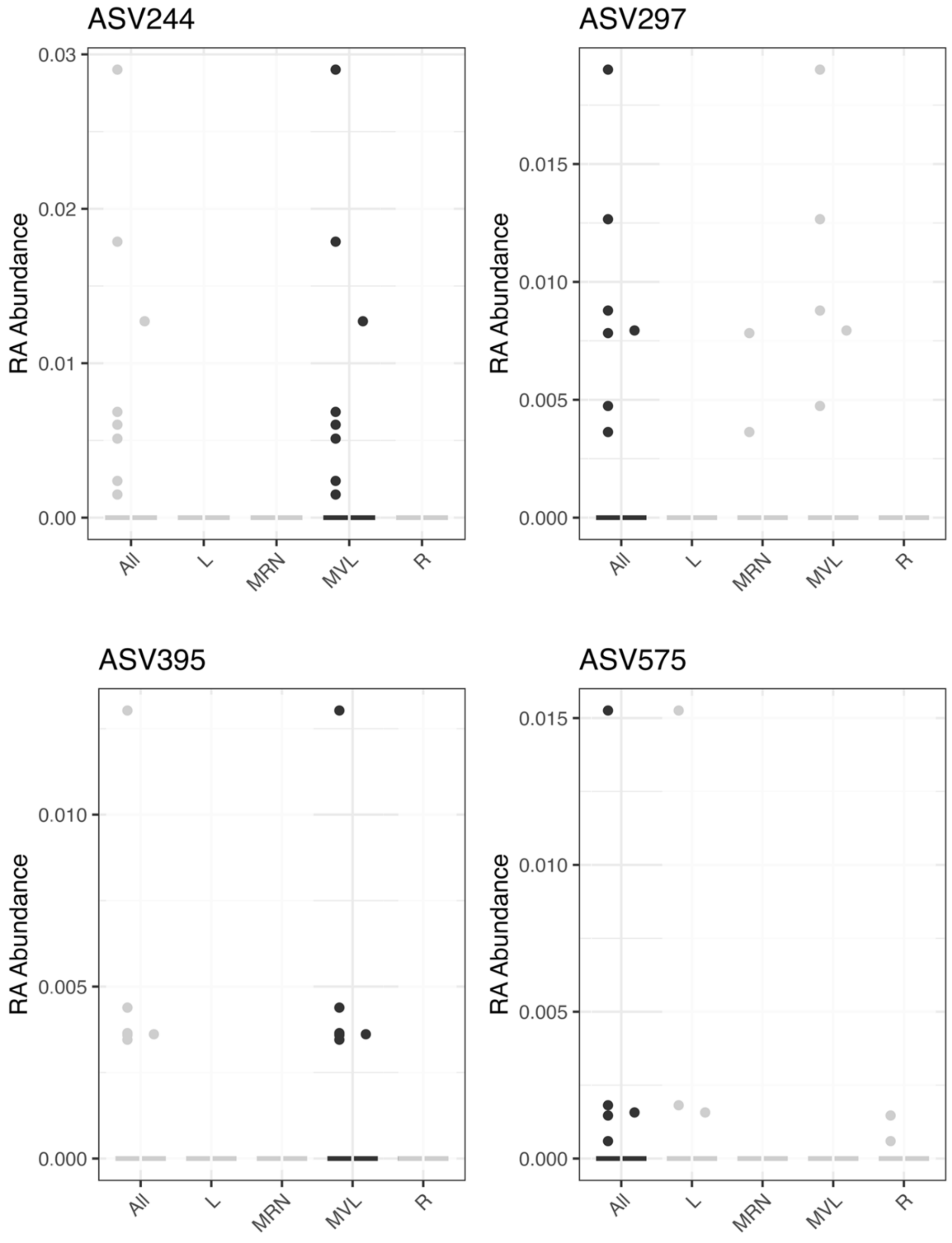

Figure S11. Boxplots of taxa with significant differential abundance between pregnant and non-pregnant ewes for ASV244, ASV297, ASV395, ASV575. Pregnancy-positive samples in blue and pregnancy-negative samples in red. The x-axis categorizes samples by herd level. Saturated colors represent groups with statistically significant differences; desaturated colors are used for groups without significant differences.

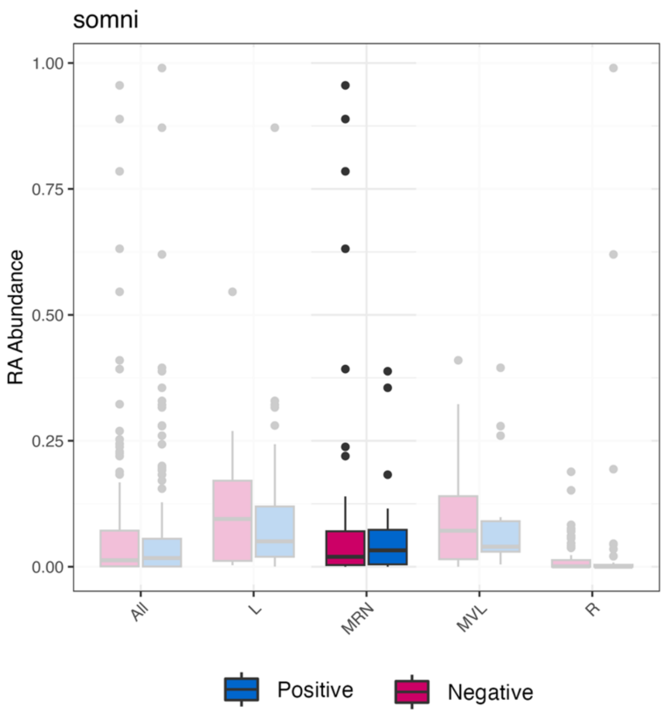

Figure S12. Boxplots of taxa with significant differential abundance between pregnant and non-pregnant ewes for the species *Histophilus somni*. Pregnancy-positive samples in blue and pregnancy-negative samples in red. The x-axis categorizes samples by herd level. Saturated colors represent groups with statistically significant differences; desaturated colors are used for groups without significant differences.

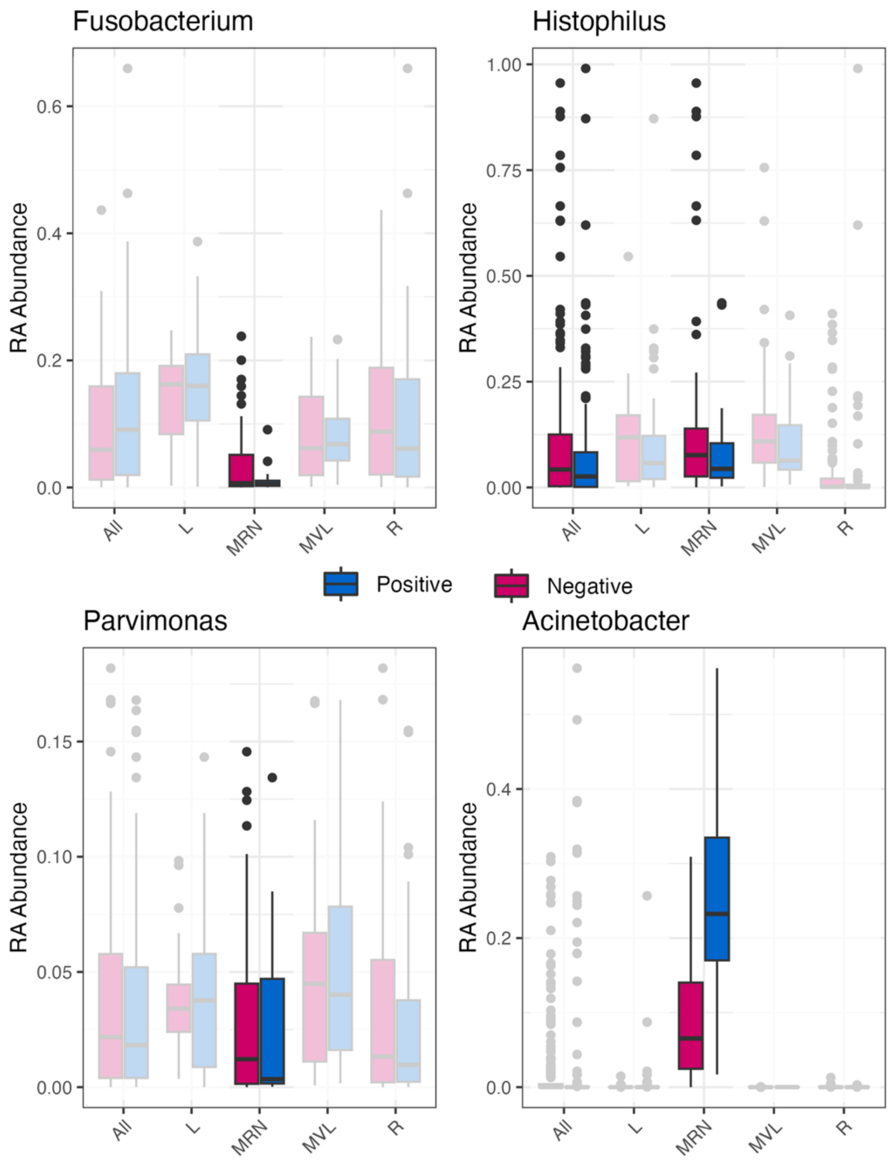

Figure S13. Boxplots of taxa with significant differential abundance between pregnant and non-pregnant ewes for the genus *Fusobacterium, Histophilus, Parvimonas,* and *Acinetobacter*. Pregnancy-positive samples in blue and pregnancy-negative samples in red. The x-axis categorizes samples by herd level. Saturated colors represent groups with statistically significant differences; desaturated colors are used for groups without significant differences.

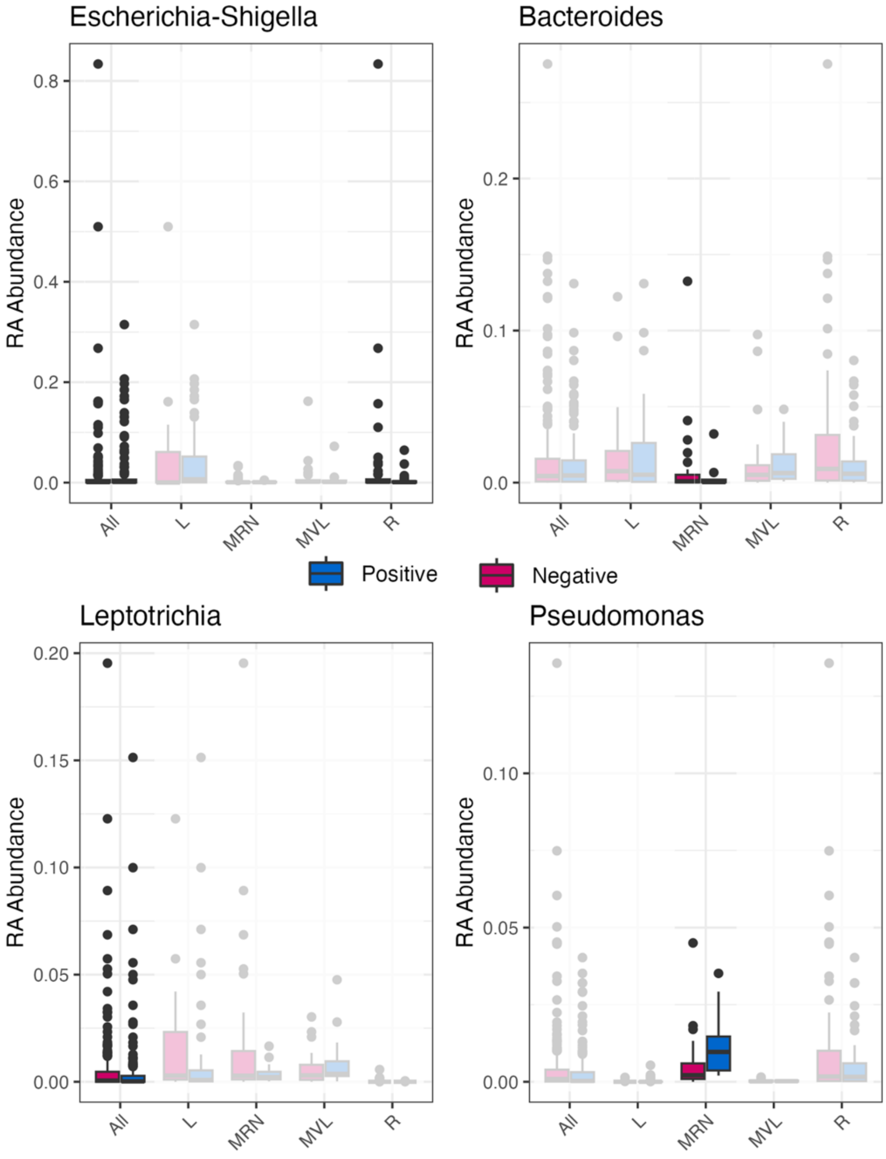

Figure S14. Boxplots of taxa with significant differential abundance between pregnant and non-pregnant ewes for the genera *Escherichia-Shigella, Bacteroides, Leptotrichia,* and *Pseudomonas.* Pregnancy-positive samples in blue and pregnancy-negative samples in red. The x-axis categorizes samples by herd level. Saturated colors represent groups with statistically significant differences; desaturated colors are used for groups without significant differences.

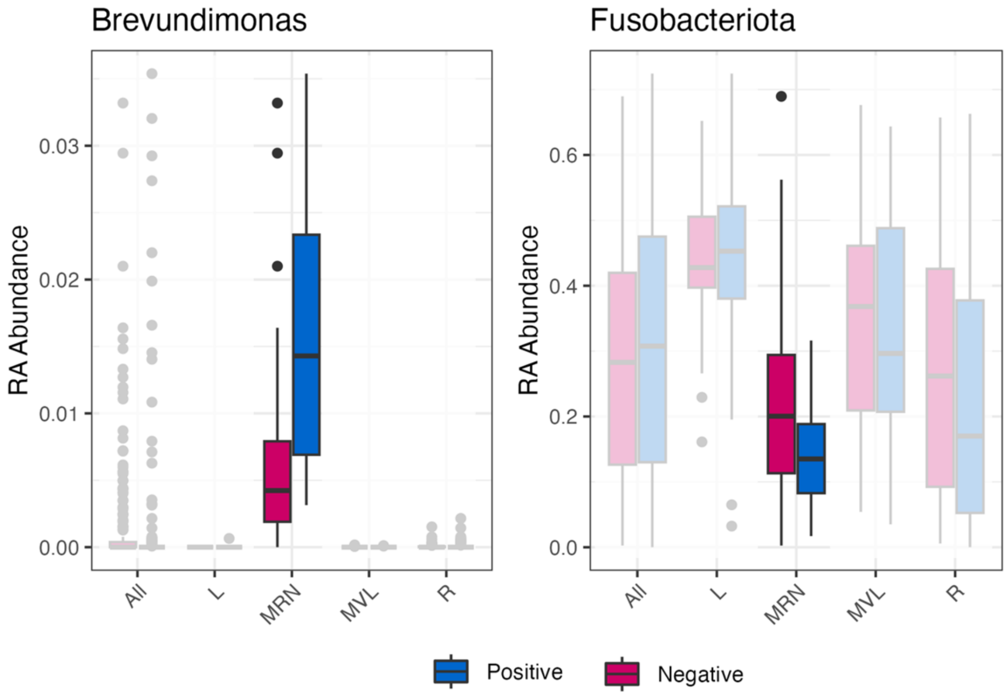

Figure S15. Boxplots of taxa with significant differential abundance between pregnant and non-pregnant ewes for the genera *Brevundimonas* in the left and the phylum *Fusobacteriota* in the right. Pregnancy-positive samples in blue and pregnancy-negative samples in red. The x-axis categorizes samples by herd level. Saturated colors represent groups with statistically significant differences; desaturated colors are used for groups without significant differences.

Table S1. Comparison between the assignment of the most significant ASVs for pregnancy by the Silva databases and the NCBI databases.

	SILVA data base		NCBI data base		
**ASV**	**Tax level**	**Tax**	**Scientific Name**	**PI %**	**Accession Number**
**ASV132**	Family	Pasteurellaceae	Actinobacillus seminis	99.53	NR_042872.1
**ASV395**	Family	Pasteurellaceae	Actinobacillus seminis	99.53	NR_042872.1
**ASV227**	species	A. pluranimalium	A. pluranimalium	100	HG423363.1
**ASV51**	Genus	Bacteroides	Bacteroides pyogenes	100	MT271930.1
**ASV17**	Genus	Escherichia-Shigella	Escherichia coli K-12	100	6XE0_W
**ASV240**	Genus	Histophilus	Histophilus somni	99.77	CP042991.1
**ASV297**	Genus	Histophilus	Histophilus somni	99.53	CP042991.1
**ASV40**	Genus	Escherichia-Shigella	Shigella sonnei	100	CP055292.1
**ASV142**	Genus	Escherichia-Shigella	Shigella sonnei	99.77	CP055292.1
**ASV168**	Genus	Corynebacterium	Uncultured Corynebacterium	99.03	FJ467410.1
**ASV66**	Genus	Porphyromonas	Uncultured Porphyromonas	100	GU905895.1
**ASV65**	Genus	Staphylococcus	Uncultured bacterium	100	HM828039.1
**ASV91**	Genus	Fusobacterium	Uncultured bacterium	100	JN857713.1
**ASV133**	Genus	Aerococcus	Uncultured bacterium	100	MF113565.1
**ASV221**	Genus	Campylobacter	Uncultured bacterium	97.02	KC936904.1
**ASV575**	Genus	Fusobacterium	Uncultured bacterium	99.51	JN857713.1
**ASV44**	Genus	Fusobacterium	Uncultured bacterium	99.51	KR514438.1
**ASV122**	Genus	Oceanivirga	Uncultured bacterium	97.04	JQ205915.1
**ASV83**	Genus	Phorimonas	Uncultured bacterium	99.76	OK049277.1
**ASV33**	Genus	Aerococcus	Uncultured bacterium	97.78	OK048949.1
**ASV159**	Genus	Fusobacterium	Uncultured bacterium	99.26	KR514438.1
**ASV29**	Genus	Leptotrichia	Uncultured bacterium	98.02	OK048932.1
**ASV244**	Genus	Mageeibacillus	Uncultured bacterium	99.75	OK037716.1
**ASV61**	Family	Weeksellaceae	Uncultured bacterium	95.97	GU905654.1
